# Ultrasound-mediated delivery and distribution of polymeric nanoparticles in the normal brain parenchyma of a metastatic brain tumour model

**DOI:** 10.1371/journal.pone.0191102

**Published:** 2018-01-16

**Authors:** Habib Baghirov, Sofie Snipstad, Einar Sulheim, Sigrid Berg, Rune Hansen, Frits Thorsen, Yrr Mørch, Catharina de Lange Davies, Andreas K. O. Åslund

**Affiliations:** 1 Department of Physics, The Norwegian University of Science and Technology (NTNU), Trondheim, Norway; 2 SINTEF Materials and Chemistry, Trondheim, Norway; 3 SINTEF Medical Technology, Trondheim, Norway; 4 Department of Circulation and Medical Imaging, The Norwegian University of Science and Technology (NTNU), Trondheim, Norway; 5 Molecular Imaging Center and Kristian Gerhard Jebsen Brain Tumour Research Centre, Department of Biomedicine, University of Bergen, Bergen, Norway; Case Western Reserve University, UNITED STATES

## Abstract

The treatment of brain diseases is hindered by the blood-brain barrier (BBB) preventing most drugs from entering the brain. Focused ultrasound (FUS) with microbubbles can open the BBB safely and reversibly. Systemic drug injection might induce toxicity, but encapsulation into nanoparticles reduces accumulation in normal tissue. Here we used a novel platform based on poly(2-ethyl-butyl cyanoacrylate) nanoparticle-stabilized microbubbles to permeabilize the BBB in a melanoma brain metastasis model. With a dual-frequency ultrasound transducer generating FUS at 1.1 MHz and 7.8 MHz, we opened the BBB using nanoparticle-microbubbles and low-frequency FUS, and applied high-frequency FUS to generate acoustic radiation force and push nanoparticles through the extracellular matrix. Using confocal microscopy and image analysis, we quantified nanoparticle extravasation and distribution in the brain parenchyma. We also evaluated haemorrhage, as well as the expression of P-glycoprotein, a key BBB component. FUS and microbubbles distributed nanoparticles in the brain parenchyma, and the distribution depended on the extent of BBB opening. The results from acoustic radiation force were not conclusive, but in a few animals some effect could be detected. P-glycoprotein was not significantly altered immediately after sonication. In summary, FUS with our nanoparticle-stabilized microbubbles can achieve accumulation and displacement of nanoparticles in the brain parenchyma.

## Introduction

Treatment of many brain diseases is severely hampered by limited access of drugs to damaged tissues; this problem is particularly salient in brain cancer where chemotherapeutic treatment of primary brain malignancies as well as metastatic brain tumours has so far shown only minimal effects on tumour growth and patient survival [1]. One of the obstacles hindering successful treatment is the blood-brain barrier (BBB)—a dynamic interface that protects the brain’s internal milieu and filters out 98% of small molecular drugs (about 400–500 Da) and all large molecular drugs [2–4]. The passage of drugs across the BBB is blocked both paracellularly, due to tight junctions connecting endothelial cells, and transcellularly, in large part due to the action of multidrug resistance transporters such as P-glycoprotein (P-gp) located in the plasma membrane [2–3]. One of the challenges in drug delivery across the BBB is accumulation of drugs in therapeutically relevant doses in the brain. This has precluded the advancement of several promising drug candidates, e.g. paclitaxel and topotecan, to the clinic in the treatment of brain tumours because, after systemic administration, the doses required to achieve a therapeutic effect were also prohibitively toxic [1].

Drug-loaded nanoparticles (NPs) have emerged as a powerful tool to reduce drug toxicity after systemic administration [5] and provide controlled and sustained release, targeting and functionalization [6]. In the case of solid tumours, NP-based drug delivery also benefits from the enhanced permeability and retention (EPR) effect whereby NPs are retained in the tumour mass due to its leaky neovasculature and reduced lymphatic drainage [7–8]. Poly(alkyl cyanoacrylate) (PACA) NPs have been shown to be promising drug carriers due to ease of synthesis and functionalization, as well as biodegradability [9–11]. These properties have recently allowed them to reach a Phase III clinical trial (ReLive study, ClinicalTrials.gov Identifier: NCT01655693) [12]. In the case of brain diseases, however, NP transport across the BBB is complicated even more than it is with small molecular drugs. Paracellular transport across an intact BBB is virtually impossible [13], and NP size is far above the passive transcellular transport threshold. Biofunctionalization of NPs with moieties conferring BBB transport properties, as well as the use of EPR in solid brain tumours do offer benefits, but the efficiency of the former approach is highly dependent on the carrier and the transport moiety, and the use of EPR has so far produced only a modest increase in drug accumulation [14].

Focused ultrasound (FUS) in combination with microbubbles (MBs) that are normally used in diagnostic ultrasonography has been shown to open the BBB safely and reversibly [15–17]. It has been employed to transport NPs across the BBB in several studies [18–21]. The use of MBs in combination with FUS to open the BBB was first demonstrated by Hynynen et al. in 2001 [22] and is based on the volumetric oscillation of MBs in the proximity of blood vessels in the ultrasound (US) focus, causing a mechanical stress on the vessel wall and leading to enhanced extravasation. The use of MBs considerably reduced the acoustic power required for cavitation-dependent opening of the BBB by FUS alone, thereby enabling safe application of transcranial US. Our group has previously demonstrated FUS-mediated BBB opening in healthy rats using a platform consisting of poly(butyl cyanoacrylate) (PBCA) NP-stabilized gas-filled MBs [23]. The aim of the previous study was to demonstrate that FUS and the NP-stabilized MBs successfully opened the BBB safely and delivered NPs into the brain tissue. It was also noted that the fluorescent dye was released from the NPs within an hour after US treatment, as PBCA degraded rather quickly [10]. In the present work we used poly(2-ethyl-butyl cyanoacrylate) (PEBCA) NPs to stabilize the MBs, which are degraded more slowly than PBCA (unpublished results) and have a longer circulation time than PBCA NPs, 136 min [24–26]. The MBs stabilized by PBCA or PEBCA NPs have a circulation time similar to commercial MBs. Using US imaging, PEBCA NP-stabilized MBs were detected in a subcutaneous tumour for approximately 4–5 min [24].

A requirement for drug-loaded NPs to have a therapeutic effect on diseases in the brain, is that the drug reaches the diseased area, which is not a trivial task for NPs due to their size [27]. Thus in the present work, the transport of NPs into the brain parenchyma was quantified with respect to the extent of BBB opening. Furthermore, part of the brain was also exposed to acoustic radiation force (ARF) produced by high frequency, high intensity and highly focused US. ARF causes a transfer of momentum from the US wave to the propagation medium and can potentially increase the displacement of NPs in the ECM. ARF has primarily been used to improve targeting of MBs [28–29] or for improved drug delivery using drug-loaded or drug-decorated MBs [30–31]. Compared to NPs, MBs are large and highly compressible, and they therefore experience a much larger effect caused by ARF. In a limited number of studies, ARF has been used for drug delivery with liquid NPs [32–33].

Since P-gp is a key component of the BBB, restricting the passage of many small and lipophilic molecules, including drugs, by pumping them back into the bloodstream [2], it is also important to assess how FUS treatment affects P-gp in the brain. Recent studies have found that FUS induces down-regulation of P-gp *in vivo* after 24 hours [34] and after 1 hour [35]. At this time point, FUS-mediated P-gp down-regulation may involve delayed onset mechanisms, and it may also be informative to study the immediate effect of FUS on P-gp expression.

In this study, we used a novel US system that can generate FUS at two frequencies, with magnetic resonance imaging (MRI) guided selection of the treatment area. 1.1 MHz was used in combination with a PEBCA-based NP-MB platform to open the BBB and deliver the NPs into the brain parenchyma in a melanoma brain metastasis model. In addition, 7.8 MHz was employed to generate ARF and investigate whether it could push NPs through the ECM away from blood vessels. We also evaluated how FUS at 1.1 MHz affected the expression of P-gp in the brain tissue immediately and 2 h post-sonication. We verified BBB opening by contrast enhanced MRI and quantified NP extravasation and distribution in the brain parenchyma in relation to the extent of BBB opening.

## Results

### Characterization of the PEBCA NP-MB platform

The synthesized PEBCA NPs were analysed using dynamic light scattering (DLS) and had a Z-average of 274 nm and polydispersity index of 0.25 while the ζ-potential was 0 mV. The MBs had a mean size of 1.6±0.85 μm (S2 Fig) and a mean concentration of 4.5E8±1.0E8 MBs/ml.

### Development of melanoma brain metastases

**Fig 1:**
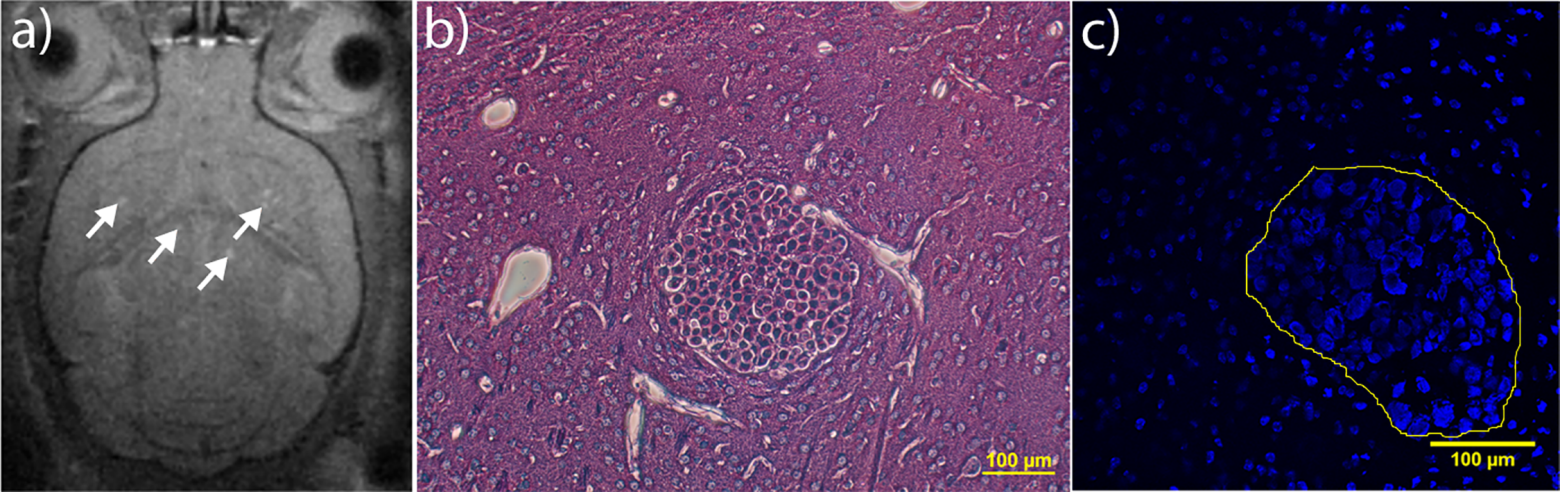
Melanoma brain metastases 4 weeks after tumour cell inoculation. Metastatic tumours are visible in a) T1 RARE MR images without contrast enhancement (some tumours are indicated by arrows), b) H&E-stained sections, as a spherical group of cells, c) CSLM with nuclei counterstaining, as a cluster of tightly packed nuclei (shown in an outline).

Melanoma brain metastases were detected using T1-RARE MRI at 28±2 days after intracardiac injection of tumour cells. Due to the metal scavenging properties of melanin [36] that is highly abundant in the tumours, the metastases were visible in MRI images without any contrast enhancement (Fig 1A). Metastatic tumours were also visible in the histological examination of formalin-fixed paraffin-embedded sections, in line with the results reported in [37] (Fig 1B), as well as in confocal laser scanning microscopy (CLSM) images of cryosections (Fig 1C). In the CLSM images and haematoxylin and eosin (H&E)-stained sections, the metastases appeared at higher cell density and with larger nuclei than normal brain cells. We did not evaluate leakiness of the metastases; however, an earlier study has shown that the percentage of leaky tumours at week 4 was about 1% [37].

### FUS-mediated BBB opening

**Fig 2:**
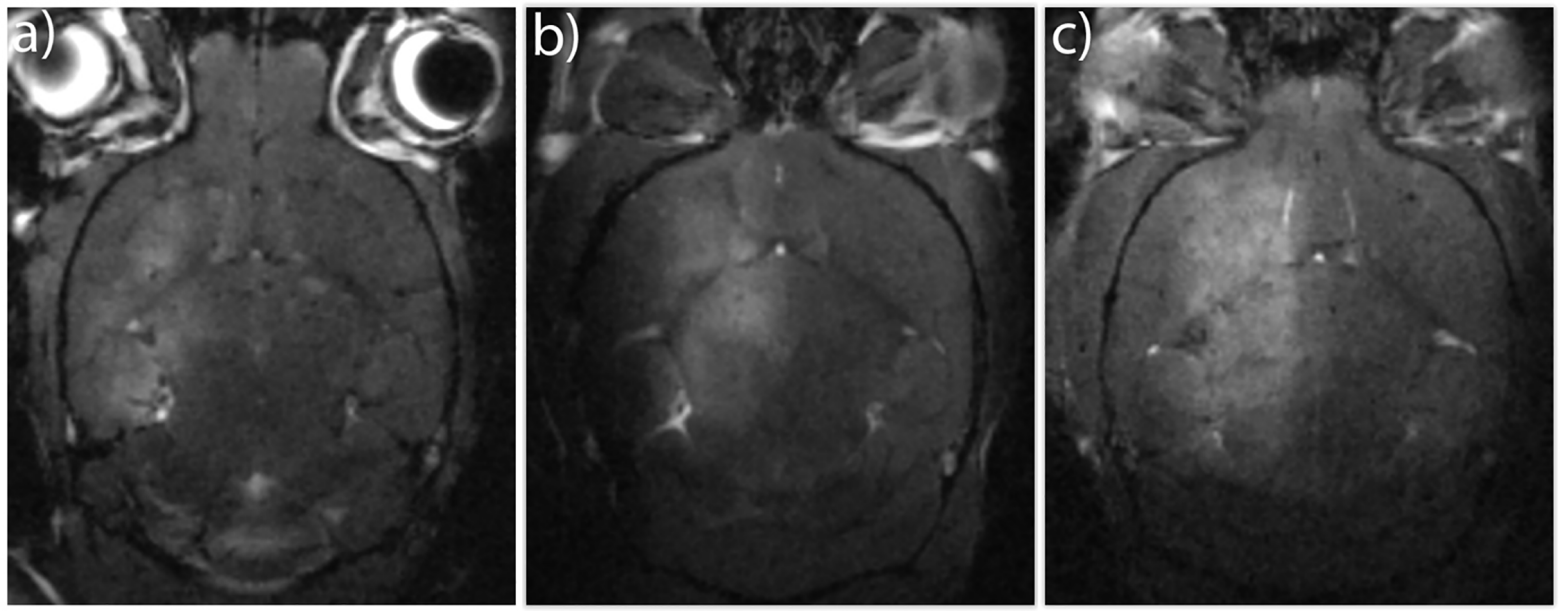
FUS-mediated BBB opening with PEBCA NP-MBs. Different extents of BBB opening are shown in panels a-c). The three brains show increasing amount of gadolinium-based contrast in the treated region, from hardly any contrast a), to large areas of high intensity contrast c) compared to the non-treated contralateral hemisphere. MRI images were normalized so that areas not exposed to FUS in different images have a comparable intensity. FUS parameters: 1.1 MHz, 5 min, pulse repetition frequency (PRF) 0.33 Hz, estimated *in situ* pressure 0.31–0.34 MPa, 10 ms burst length.

FUS-mediated BBB opening was observed in all animals in all groups, but its extent, assessed using signal intensity in T1 FLASH images, varied between the animals (Fig 2A–2C).

**Fig 3:**
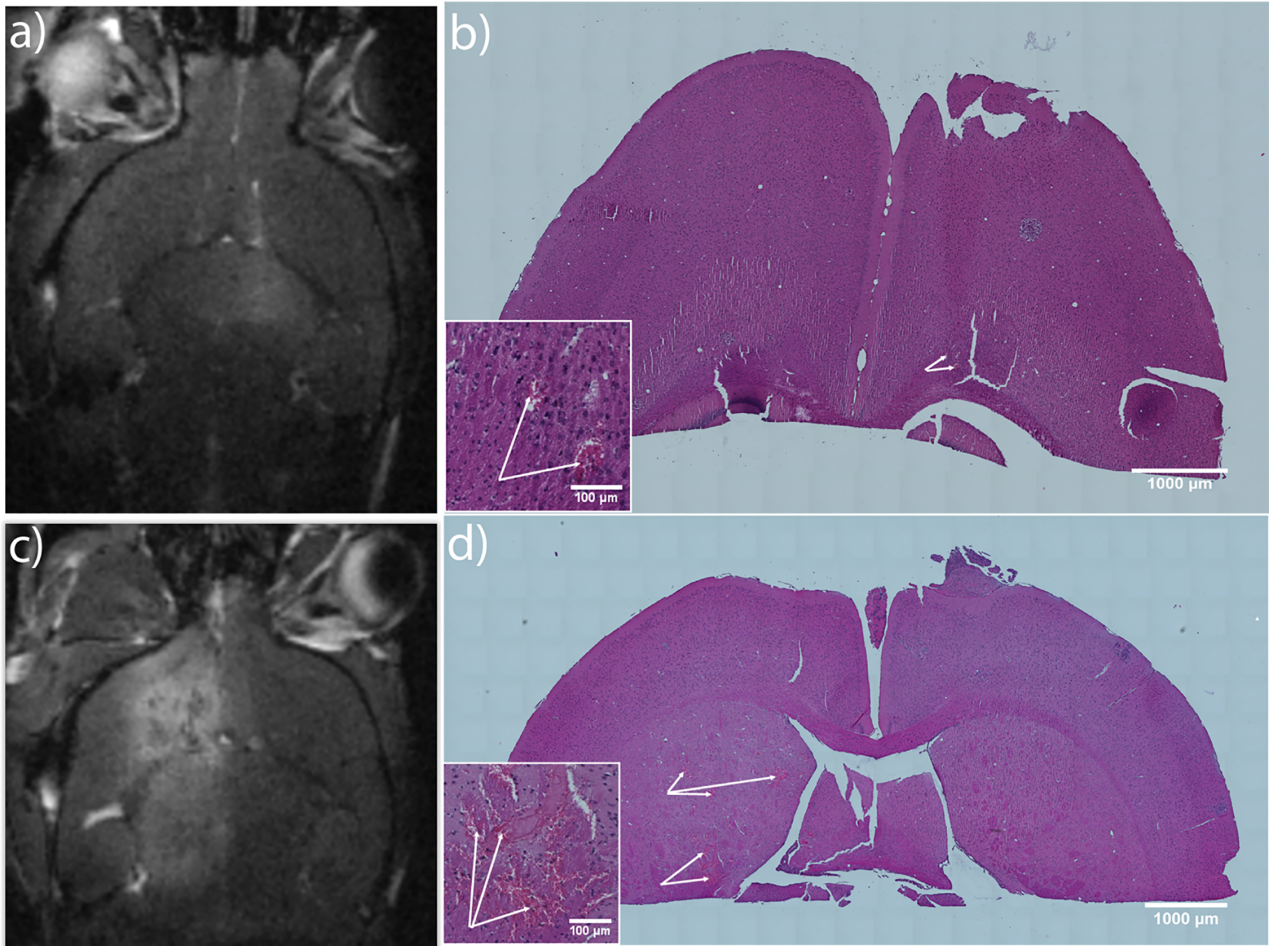
RBC extravasation in H&E-stained brain sections. a-b) MR image of a brain with a corresponding H&E-stained section with limited RBC extravasation. c-d) MR image of another brain with corresponding H&E-stained section with considerable extravasation of RBC. In b) and d), arrows show RBC extravasation and insets show zoomed areas with RBC extravasation. MRI images were normalized so that areas not exposed to FUS in different images have a comparable intensity. FUS parameters: 1.1 MHz, 5 min, pulse repetition frequency (PRF) 0.33 Hz, estimated *in situ* pressure 0.31–0.34 MPa, 10 ms burst length.

T1 FLASH images from MRI demonstrated some extent of red blood cell (RBC) extravasation. This was also evident from tile scans of histological brain sections (Fig 3). The extent of RBC extravasation varied from relatively small and localized to covering large areas of the treated hemisphere, depending on the extent of the BBB opening.

### NP transport across the BBB and distribution in the brain parenchyma and metastases

**Fig 4:**
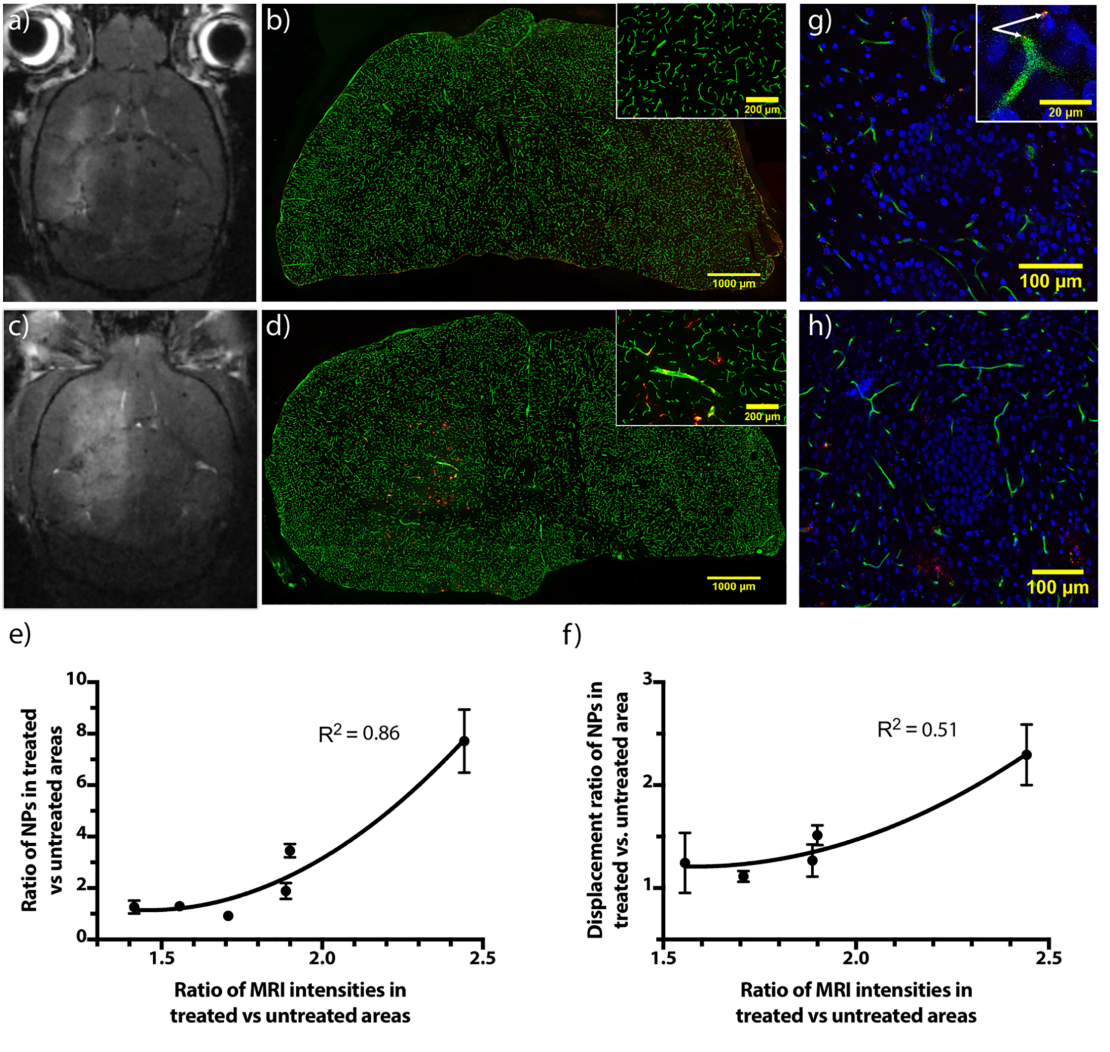
FUS-mediated NP transport across the BBB and distribution in the brain parenchyma. a) MR image of a brain with a corresponding CLSM tile scan b) showing little difference between NP amount in the treated and untreated hemispheres. c) MR image of another brain with a corresponding CLSM tile scan d) showing substantially greater differences between NP amount in the treated and untreated hemispheres. Insets in Fig 4B and Fig 4D show an enlarged image of the treated area. e) Uptake of NPs across the BBB. n = 3–5 sections per mouse. f) Displacement of NPs away from blood vessels. n = 3–5 sections per mouse. Values represent the mean of each animal, and error bars give standard error of the mean. The lines are fitted using a monoexponential function. g-h) CLSM images with tumour metastases visible as a cluster of tightly packed nuclei. Inset g) shows an enlarged area of the tumour, with NPs visible both inside and outside of a blood vessel (indicated by arrows). Colours in CLSM images are: Red–NPs, blue–nuclei, green–blood vessels. MRI images were normalized so that areas not exposed to FUS in different images have a comparable intensity.

BBB opening-dependent transport of NPs following FUS exposure was demonstrated (Fig 4A–4D). The amount of NPs entering into the brain parenchyma and the extent of NP displacement from the nearest blood vessel wall correlated with increasing MRI contrast agent intensity (Fig 4E and 4F). Up to 7.7 times more NPs (average 2.8 times) were found in the treated hemisphere compared to the untreated. The average displacement of NPs from the nearest blood vessel was up to 2.3 times higher in the treated hemisphere compared to the untreated one. Changes in the transport and distribution of NPs were minor up to a certain BBB opening extent (ratio of MRI intensities approx. 1.5–2.0), and then increased rapidly. NPs were also visible in melanoma brain metastases (Fig 4G and 4H) both inside and outside of blood vessels (shown in enlargement as an inset in Fig 4G). Fig 4 is from mice in group 2 and the sections analysed are from the part of the brain only exposed to 1.1 MHz. The transport of NPs across the BBB in animals in group 1 also increased proportionally to the extent of BBB opening (not shown). The data for individual mice are shown in S2 File.

### ARF effect on the displacement of NPs

**Fig 5:**
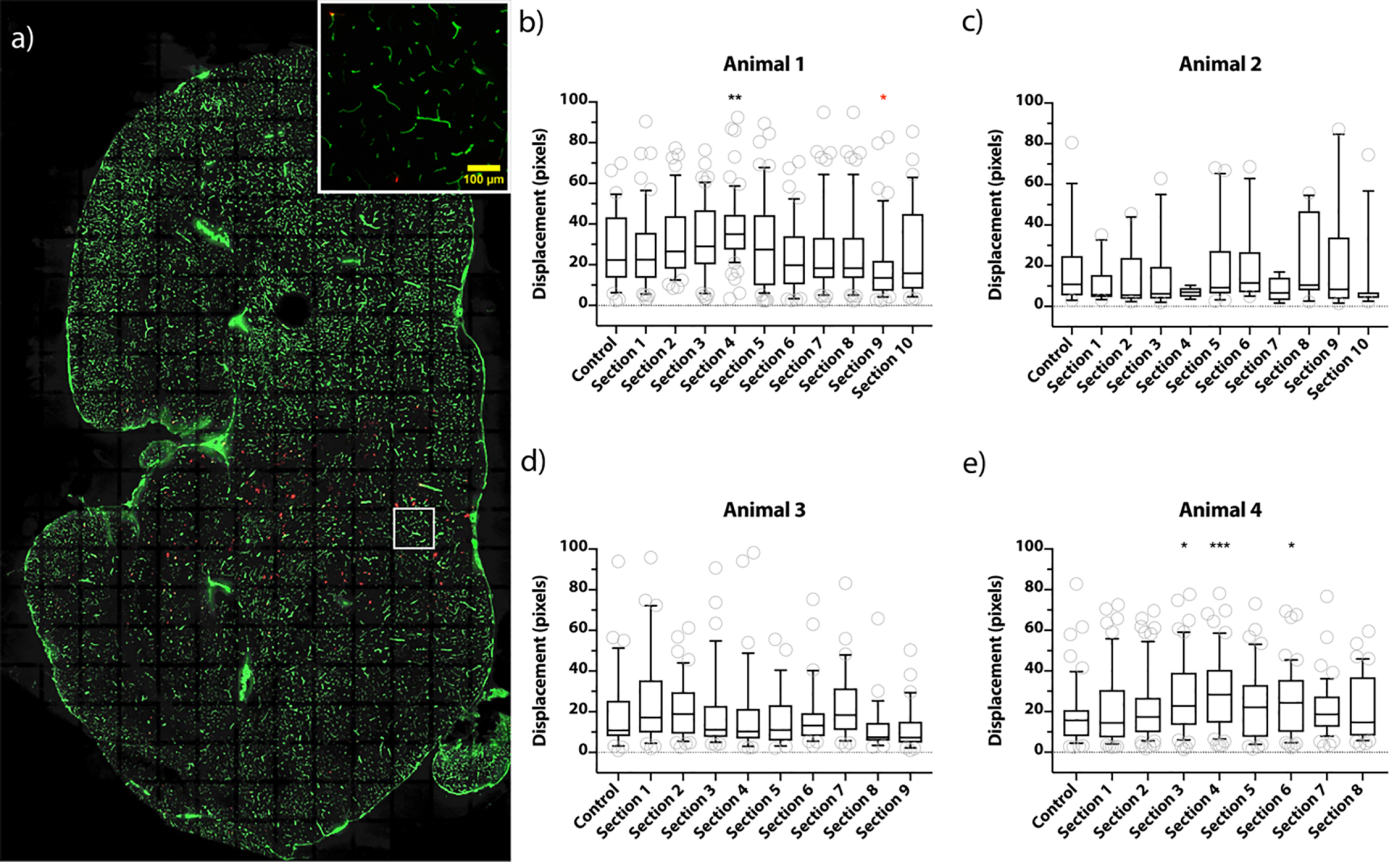
The effect of the ARF on NP displacement in the brain parenchyma. a) A tile scan of an entire brain section taken with a 20x objective. NPs are shown in red and blood vessels in green. Inset shows an individual image of the tile scan; scale bar in the inset– 100 μm. b-e) Image analysis of ARF effect from four animals. Control denotes a section from the brain area that only received 1.1 MHz FUS treatment, while numbered columns refer to sections located within and slightly extending beyond the estimate area of the ARF effect. Each column refers to one section and each circle in b-e) represents the median displacement from blood vessels of all NPs in a specific image from a section (marked with a white box in a)). Data is represented as a box-and-whisker plot where the whiskers correspond to the 10–90 percentile. 1 pixel corresponds to 0.45 μm. * p ≤ 0.05, ** p ≤ 0.01,*** p ≤ 0.001, (significantly lower than control).

To study whether ARF could increase the displacement of NP from blood vessels even further, smaller regions of brains were also treated with higher frequency and highly focused US to generate ARF. An example of a tile scan image of the entire brain is shown in Fig 5A, and the inset shows one individual image of the tile scan. A Dunn’s multiple comparison showed that some sections in animal 1 (Fig 5B) and 4 (Fig 5E) indicated enhanced displacement compared to the control, however, in animal 1 one section was significantly lower than the control. Animal 2 (Fig 5C) and 3 (Fig 5D) did not indicate any enhanced displacement. It should be noted that the tile scan in Fig 5A is shown for illustration purposes, and the actual image analysis was performed on individual images shown in the inset and the white box in the tile scan. The data for individual mice and brain sections are shown in S2 File.

### P-gp effect

**Fig 6:**
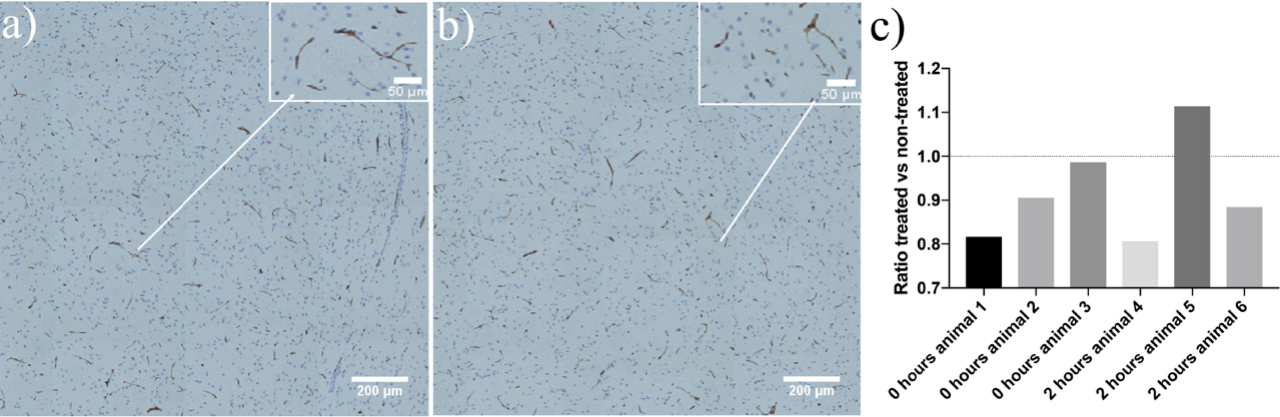
The effect of FUS-mediated BBB opening on P-gp levels immediately and 2 h after sonication. Fragment of a brain section tile scan showing P-gp staining immediately after FUS in a) treated hemisphere, b) untreated hemisphere. Enlarged area with P-gp staining are shown in insets. P-gp is shown in brown, and cell nuclei in blue. c) The ratio of P-gp expression from treated vs untreated areas.

P-gp staining in the brains after sonication is shown in Fig 6. We were not able to detect any significant change in P-gp expression comparing the treated versus untreated hemisphere at 0 or 2 h post treatment (Fig 6C). However, some animals showed a clear reduction, which could be related to the treatment. The data for individual mice and tissue sections are shown in S2 File.

## Discussion

While it is difficult to deny the potentially enormous advantages that materials science can confer to nanomedicine in preference to traditional drug formulations, the initial enthusiasm surrounding nanomedicine's applications has been called into question in light of the limited number of translational successes. In the case of solid tumours, a survey of the literature from the past 10 years has shown that solid tumours retain only 0.7% (median) of the administered NP dose [14]. The delivery of therapeutic NPs to the brain can be aided by BBB opening techniques such as FUS-mediated BBB disruption. However, even with FUS, the question is whether NP delivery to the brain parenchyma is sufficient to achieve a therapeutic effect.

In this work, the BBB was opened using FUS in combination with a novel PEBCA NP-MB platform [26]. The platform consists of PEBCA NPs sized approx. 270 nm stabilizing MBs sized 1.6 μm. Our group has previously used this platform for FUS-mediated BBB opening in healthy rats, demonstrating reversible BBB opening and the delivery of PBCA NPs into brain parenchyma [23]. PEBCA polymer was chosen in the present study, as PEBCA degrades more slowly than PBCA, which can be of therapeutic benefit. This allowed us to track intact NPs which have not released the dye. Rapidly degrading particles will release the dye and make the analysis more difficult. Degradation of PACA NPs is dependent on the length and bulkiness of the alkyl chain [10,38], and PEBCA is bulkier than PBCA due to branching alkyl chains. Another reason for choosing the PEBCA platform is the promising treatment study that was recently published [24]. It was shown that US-treatment of subcutaneous tumours using PEBCA-NP stabilized MBs (with cabazitaxel encapsulated in the NP) induced complete remission in the animals.

In the present work, we opened the BBB by applying FUS at an estimated *in situ* pressure of 0.31–0.34 MPa in order to obtain sufficient NP extravasation for quantitative measurement of NP distribution. Although BBB opening with 1.1. MHz FUS was done using the same US parameters in all animals, the extent of opening, as judged by contrast enhancement in MRI, varied. This variability could be attributed to angle and height differences in skull positioning during the FUS treatment, batch-to-batch variability in NP-stabilized MBs or a combination of these factors. The NP transport across the BBB correlated with the extent of BBB opening. In addition, the extent of BBB opening also correlated with RBC extravasation as observed in histological sections. However, a certain amount of RBC in the tissue might be clinically acceptable when treating terminal diseases such as brain cancer, especially considering that their current treatment strategies often involve surgical resection and/or intracranial administration of drugs, i.e. procedures that are much more invasive.

While some NPs were shown to be delivered to melanoma brain metastases, the delivery was restricted as compared to the surrounding brain tissue. This is consistent with the properties of this melanoma metastasis model at week 4 post-inoculation. As observed in Thorsen et al. [37], at this time point the mean vascular area fraction in the tumours is lower than in the normal brain. This is also evident in our CLSM images. It is therefore likely that reduced vascularization would diminish NP delivery into the tumour tissue in our study. As the diseased tissue gets increasingly vascularized, the extent of NP delivery to metastases in our model would probably increase.

Assuming mainly paracellular transport across the BBB, NP size can be a critical factor. Size dependence of FUS-mediated NP transport has been investigated in several works. For instance, Choi et al. used fluorescently labelled dextrans of 3, 70 and 2000 kDa and reported that the molecular weight of dextrans that could be delivered to the brain parenchyma using FUS in combination with SonoVue MBs (0.57 MPa corresponding to MI of 0.46) was between 70 kDa and 2000 kDa, and that 2000 kDa dextrans (54.4 nm) could not be delivered [39]. However, in a subsequent study from the same group, 2000 kDa dextrans co-administered with MBs (Definity) 6 seconds after the start of sonication were delivered into brain parenchyma at 0.84 MPa [40]. In a study using FUS exposure parameters that were similar to the ones used by Choi et al. [39] and NPs injected after sonication, the maximum gap between endothelial cells caused by FUS exposure was found to be close to 65 nm [41]. The authors suggested the transport of large objects >100 nm to be difficult, assuming purely paracellular mode of transport. However, there are several studies demonstrating that NPs with diameters in the range of 60–120 nm have been delivered into the brain parenchyma using FUS-mediated BBB opening. For instance, biodegradable polymeric NPs sized 60 nm and 75-nm could be delivered across the BBB after FUS exposure when albumin MBs, similar to Optison, were co-injected with the NPs immediately before FUS exposure [18]. The extent of NP transport across the BBB was shown to depend on the acoustic pressure. Mead et al. [21], have found that DNA-bearing NPs sized 100 nm and co-injected with the same MBs as used in Nance et al. [18] were delivered into the brain parenchyma after FUS exposure with 0.6 MPa and a duration of 2 minutes. Diaz et al. [19] reported brain delivery of 50 nm and 120 nm gold NPs after FUS-mediated BBB opening with approximately 0.23 MPa when NPs were injected 8 minutes prior to sonication. An overview of these studies indicates that the extent of brain delivery is dependent on numerous variables (properties of NPs, concentration and circulation time of MBs, FUS exposure parameters etc.), of which the most important ones appear to be the size of the NPs, the mechanical index and the presence of NPs in blood during sonication. In our study, the NPs, being part of the NP-MB platform, were present in the bloodstream during FUS-mediated BBB opening and were delivered more effectively compared to the control, despite being significantly larger than the NPs discussed above. NPs lining the shell of the MBs, moreover, were in the immediate proximity to the MBs during FUS-induced cavitation of the latter, which has been shown to improve NP delivery to tumours [42] and may facilitate their transport across the BBB.

The FUS exposure performed in the presence of circulating NP-MBs, which induce cavitation, will probably produce biomechanical effects on the blood vessel wall. We demonstrated that in addition to the increased transport of NPs across the BBB, FUS at 1.1 MHz increases NP distribution in the brain parenchyma, i.e. displacement from blood vessels, although the exact mechanism is unclear. From *in vitro* measurements, we know that the majority of the PEBCA NP-MBs will collapse at the *in situ* acoustic pressure used in the current experiment. During sonication, the MBs will be pushed towards the endothelial cell lining, and it is well known that under asymmetric boundary conditions the bubble collapse will also be asymmetrical [43], resulting in a liquid jet impinging on, and potentially penetrating, the solid boundary (i.e. the endothelial lining). In addition to BBB opening, this process may also facilitate NP distribution in the brain tissue, which is a considerable advantage given that passive diffusion of NPs in the ECM can be very restricted, especially in the case of large NPs [18].

The effect of the ARF could potentially further increase NP displacement from blood vessels, improving their distribution in the brain. Due to lower acoustic absorption, the intensities required to directly affect liquid and solid NPs using ARF are higher compared to gas-filled particles. However, high intensity ARF can also act on the bulk fluid, which can induce acoustic streaming that can indirectly affect the NPs and increase NP displacement in the brain parenchyma [33]. The effect of ARF NPs was limited in this study, and should be considered an incentive for further studies. The reason could be that the method used to assess the effect of ARF had some inherent limitations. As this method was based on image analysis, any NP displaced from a given blood vessel beyond the distance half way to the neighbour vessel would be registered as originating from that vessel. This makes it challenging to isolate the effect of the ARF if it is combined with the effect of another exposure (at 1.1 MHz) that already causes displacement of NPs. Moreover, the variation in the effect of ARF might be due to analysing not only tissue in the small focal region of the high frequency transducer.

Down-regulation of P-gp by US has been observed in glioma cells [44] and, in recent studies, FUS caused localized P-gp down-regulation at the BBB 24 h [34] and 1 h [35] post sonication.

In our study, no significant effects of FUS on P-gp levels were observed immediately or 2 h after sonication. Nonetheless, two of the animals (animal 1 and 4) showed a reduction that was lower than for the other animals and this could be cause by the FUS treatment. Since no MRI was acquired after sonication in the 0 h group, the extent of BBB opening could not be assessed. For the 2h time point, animal 5 showed minor opening and animal 4 and 6 showed moderate opening.

In conclusion, we have shown that our PACA NP-stabilized MB platform designed for the application of FUS in drug delivery can transport large NPs across the BBB and achieve their distribution in the brain tissue, and that the NP transport depends on the extent of FUS-mediated BBB opening. The ARF study needs to be followed up in order to make solid conclusions regarding its effect on NP displacement. Overall, our results show that while FUS-mediated BBB opening, like any generic BBB opening technique, may impose size restrictions on NP extravasation, combining NPs and MBs into a single unit such as the one used in our study can achieve a substantial increase in the transport and distribution of NPs up to 270 nm in the brain parenchyma.

## Methods

### Nanoparticles and microbubbles

PEBCA NPs were synthesized by the miniemulsion polymerization method as described and used previously to make PBCA NPs [26]. In the oil phase, the monomer (EBCA, Henkel Loctite) was mixed with a co-stabilizer (Miglyol 810N, Cremer), the fluorescent dye NR668 (modified Nile red [45]), V-65 (2,2'-Azobis(2,4-dimethylvaleronitrile, 1wt%, Wako) and methanesulphonic acid (MSA, 0.1wt%, Sigma-Aldrich). The water phase contained the PEG-stabilizers Kolliphor HS15 (Sigma-Aldrich) and Brij L23 (Sigma-Aldrich), and 0.1M HCl. The phases were mixed and sonified on ice for 4 minutes using an ultrasonic homogenizer (Branson). The resulting emulsion was left to polymerize for 1 h at ambient temperature before adjusting the pH to 5. The polymerization was continued for another 2 h at ambient temperatures followed by 8 h at 50°C. Finally, the NPs were dialyzed extensively against 1 mM HCl (MWCO 100,000) and centrifuged at 3000 rpm for 15 minutes to remove unwanted aggregates. Size and ζ-potential were measured in phosphate buffer (pH 7) using DLS (Zetasizer Nano ZS, Malvern).

MBs were made following the procedure described previously [23,26]. In brief, NPs were diluted to a concentration of 10 mg/ml in 5 mg/ml casein (Sigma-Aldrich) and 1x phosphate-buffered saline (PBS). The solution was then saturated with perfluoropropane gas (Fluorochem) and mixed violently for 2 minutes before using an Ultra Turrax (ICA Werke) to create NP-stabilized MBs with a gas core. Size and concentration of MBs was analysed using a cell counting chamber slide (Countess, Thermo Fisher). The size distribution is shown in S2 Fig. Eight positions on the slide were imaged using bright field microscopy at 20x magnification, and the size distribution and concentration was calculated using ImageJ 1.48j [46] and Excel 2010.

### Cells and animals

The H1_DL2 cell line used in this study is based on the H1 cell line isolated from a patient biopsy of human melanoma brain metastases as described previously [47]. The H1_DL2 cell line was developed by transducing H1 cells with two lentiviral vectors encoding Luciferase and a GFP variant of Dendra [37]. The cells were cultured in Dulbecco’s modified Eagle’s medium supplemented with 10% fetal bovine serum (Gibco), 2% L-glutamine (Thermofisher Scientific), and MEM Non-Essential Amino Acids Solution (100X) (Thermofisher Scientific) diluted 1:25. The growth medium was exchanged twice a week.

Female NOD/SCID mice (eight weeks of age, 18–22 g of weight) were purchased from Harlan. The mice were housed in individually ventilated cages (Techniplast). In accordance with the recommendations set forth by the Federation of European Laboratory Animal Science Associations, animal housing conditions were free of specific pathogens. The mice were provided with sterile water and food ad libitum. All animal procedures were approved by the Norwegian National Animal Research Authorities, Mattilsynet, https://www.mattilsynet.no/.

### Intracardiac injection of tumour cells

Before and during tumour cell inoculation, the animals were anesthetized with 3% isoflurane in oxygen (flow 2 l/min). 5×10^5^ of H1_DL2 cells in 0.1 ml PBS were injected into the left cardiac ventricle of the mice using a 30G insulin syringe (Omnican50, B. Braun Melsungen AG). The injection was guided by ultrasonography using a Vevo 2100 System with an MS200 transducer (Fujifilm Visualsonics). The success rate of US-guided intracardiac injection of melanoma cells (as evaluated by eventual tumour development) was 100%. The metastases were allowed to develop for 28±2 days. The procedure of intracardiac injection of tumour cells is shown in S1 Video. After the intracardiac injection, the mice received a subcutaneous injection of temgesic (Reckitt Benckiser) (0.05 mg/kg) for prolonged analgesia.

### Magnetic resonance imaging

MRI was performed using a 7.05 T horizontal bore magnet (Biospec 70/20 Avance III, Bruker Biospin). The mouse was anesthetized using a subcutaneous injection of a 2:1:2:5 mixture of fentanyl (Actavis Group hf), medetomidine (Orion Pharma), midazolam (Accord Healthcare Limited) and water at a dose of 100 μl per 10 g of body weight, and cannulated in the tail vein with a 24G catheter (BD Neoflon, Becton Dickinson Infusion Therapy). Temperature and respiration rate were monitored using rectal temperature and pressure-sensitive probes (SA Instruments), respectively. The temperature of the animal was maintained at 37°C. Once the mouse was placed in the MR scanner, the coils were tuned and matched, followed by acquisition of a localizer scan. The following MR sequences were used for pre- and post-treatment images: T1-RARE for detecting melanoma brain metastases as described previously [37], and T1 Fast Low Angle Shot (FLASH) for detecting BBB disruption based on the extravasation of a gadolinium-based contrast agent Omniscan (GE Healthcare AS, 0.5 mmol/kg, 1 ml/kg) and for detection of haemorrhages. All MR sequences had the same geometry with FOV of 40 × 27 mm, matrix size (MTX) of 200 × 135, and 12 slices at 1 mm. MRI parameters were set using Bruker Paravision v6.

### Characterization of US attenuation through the skull

The RK-100 system for MRI guided FUS treatments (FUS Instruments) including a custom made dual frequency transducer was used in the experiments. The transducer had an inner circular part (7.8 MHz) and an outer ring (1.1 MHz), with apertures of 39 mm and 52 mm, respectively. Both were focused at 60 mm. The -3 dB beam width and length in the focus were 1.6 and 47.2 mm respectively for 1.1 MHz and 0.5 and 5.4 mm respectively for 7.8 MHz. The acoustic attenuation through the skull bone was measured at both frequencies on harvested mouse skulls from animals of similar size as the ones used for BBB disruption experiments. The measurements were conducted as described by Åslund et al. [23] and were performed with pulse lengths of 20 μs (22 cycles at 1.1 MHz and 155 cycles at 7.8 MHz). At 1.1 MHz the acoustic pressure was attenuated between 10 and 17%, and at 7.8 MHz the attenuation was between 70 and 75%, depending on the angular position of the skull with respect to the incoming wave.

### FUS treatments

The mice were divided into four groups. All groups were treated with BBB-opening FUS (1.1 MHz, 5 min, pulse repetition frequency (PRF) 0.33 Hz, estimated *in situ* pressure 0.31–0.34 MPa, 10 ms burst length). Additionally, group 2 was treated with ARF (7.8 MHz, 60 min, PRF 1 Hz, estimated in situ pressure of 0.75–0.9 MPa, 5 ms burst length). The frequency (7.8 MHz) was chosen to significantly enhance tissue attenuation (compared to 1.1 MHz). The rationale behind the duty cycle (pulsing scheme) was a trade-off between enhancing the impulse delivered by the radiation force and limiting the local temperature increase.

- Group 1 (n = 3): BBB-opening treatment, euthanized 2 h after treatment start. Used for the assessment of FUS-mediated NP transport across the BBB, NP distribution in the brain parenchyma and the 2 h time point for P-gp analysis.
- Group 2 (n = 6): BBB-opening and ARF treatment, euthanized 2 h after treatment start. Used for investigating the effect of the ARF (see S1 Fig).
- Group 3 (n = 2) BBB-opening treatment, euthanized 2 h after treatment start. Used for haematoxylin and eosin staining.
- Group 4 (n = 3): BBB-opening treatment, euthanized immediately post treatment. Used to investigate the immediate effect of FUS-mediated BBB disruption on P-gp in the brain.

Before FUS treatment, the animals were anesthetized as described above in 2.4. After the heads were shaved, a depilatory cream was applied to remove the remaining hair. The mice were placed in the MR bed, and the bed was placed in the scanner. Two different MRI scans were acquired, T1-RARE without Omniscan and T1-FLASH with Omniscan. The T1-FLASH sequence was used for treatment planning according to S1A Fig. A grid of 6x2 circular treatment locations (beam width: 1.6 mm per location) was used to open the BBB in the left hemisphere in all animals, except for one animal in Group 2 that was treated in the right hemisphere since no metastases were visible in the left hemisphere. The contralateral hemisphere was used as non-treated control. The animal was placed above the transducer, and Omniscan (1 mL/kg) and NP-MBs (5 mL/kg) were injected sequentially. The FUS treatment (1.1 MHz) was initiated upon injection of the NP-MBs using a RK-100 system for MRI-guided FUS-mediated BBB disruption. After the treatment, the animal was scanned with MRI (T1-FLASH) for verification of BBB opening as well as indications of haemorrhage. The post-treatment T1-FLASH was used for ARF treatment planning and a grid of 2x2 treatment locations (beam width: 0.5 mm per location) was defined in the area where BBB opening had been successful (S1B Fig). ARF treatment was initiated 30 min after BBB opening treatment start. 2 h post anesthetization a new anaesthetic dose was injected subcutaneously at half the initial dose to maintain deep anaesthesia. 2 h post BBB opening, animals in Group 1 and Group 2 were injected with DyLight 649-labeled Lycopersicon esculentum (tomato) lectin (Vector Laboratories Inc) (5 mg/kg) to label the vasculature. Five minutes later, the mice were euthanized by cervical dislocation. The brains were removed, divided according to S1D Fig, embedded in Tissue-Tek CRYO-OCT Compound (Sakura), and frozen in a mixture of 2-methylbutane and dry ice. Animals in Group 3 were euthanized by i.v. injection of pentobarbital (100 mg/kg) followed by intracardiac perfusion with PBS and 4% paraformaldehyde. The brains were removed and submerged in 10% formalin for at least 24 h before paraffin embedding and sectioning into 4 μm sections for H&E staining. Animals in Group 4 were euthanized by cervical dislocation immediately after the FUS treatment. The brains were removed without prior injection of Lycopersicon esculentum (tomato) lectin and frozen as described above. Brains in Group 2 were sectioned as described in S1D Fig, and brains in Group 1 were sectioned axially; in both groups, the brains were cut into 4 and 20 μm cryosections. Frozen sections from Group 1 and 4 were stained using an anti-P glycoprotein antibody (EPR10364-57, Abcam, 1:200 dilution) followed by incubation with HRP (horseradish peroxidase) Rabbit EnVision–Polymer and DAB+ (3,3'-Diaminobenzidine) Chromogen (both from DAKO).

### Confocal laser scanning microscopy

For quantification of NR668-containing PEBCA NPs in the brain tissue and assessment of the ARF effect, cryosections were thawed for approx. 10 min and tile scans of entire brain sections were obtained using a Leica TCS SP8 CLSM (Leica Microsystems) without mounting. Tile scans were acquired using 10x/0.45 and 20x/0.75 air objectives. For images taken with the 10x/0.45 objective, the following parameters were used: image size: 1024x1024 pixels, zoom factor: 1.5, pixel size: 758 nm. For images taken with the 20x/0.75 objective, the following parameters were used: image size: 1280x1280 pixels, zoom factor: 1.5, pixel size: 454 nm. A white light laser was used to excite NR668 (excitation wavelength 535 nm, emission wavelength range 560–630 nm) and DyLight 649 (excitation—649 nm, emission wavelength range 660–710 nm). In order to visualize melanoma brain metastases with tightly packed nuclei, some sections were mounted with Vectashield mounting medium (Vectorlabs) containing 4',6-diamidino-2-phenylindole (DAPI) as a nuclei counterstain. DAPI was excited using a 405 nm laser with a detection of 416–468 nm. H&E- and P-gp stained sections were imaged using an LSM 800 (Zeiss) CLSM with 20x air objective in bright field mode.

### Image analysis

MR images were processed using Sante DICOM Viewer v. 5.04 and ImageJ 1.49k. Processing and analysis of CLSM images were performed, depending on the required task, by ImageJ 1.49k, Icy v.1.8.6.0 [48] or CellProfiler 2.1.1 [49]. ImageJ and CellProfiler were collectively used for image conversion, image stitching, calculation of intensity ratios in MR images, background removal, filtering, thresholding, quantification of NP count and quantification of NP displacement, Icy—for k-means thresholding and distance transform used in algorithm validation and comparison of different thresholding strategies. A more detailed description of image analysis is provided in S1 Document.

For the quantification of the ARF effect, NP displacement from blood vessels was analysed in those parts of brain sections that corresponded to the hemisphere exposed to FUS at 1.1 MHz and 7.8 MHz. The area of ARF exposure was estimated using T1 Flash MR images as shown in S1C Fig, and the corresponding brain sections were selected appropriately to cover and slightly extend beyond this area. Control sections for ARF corresponded to areas only exposed to FUS at 1.1 MHz located approximately 3 mm from the estimated ARF exposure area, in order to minimize the effect of local inhomogeneity in BBB opening. The extent of BBB opening in T1 FLASH MR images was assessed by determining the ratio of intensities in the treated and untreated brain hemispheres.

For quantification of P-gp, histological sections of the brains Tile scans were acquired for the entire sections. Images were analysed in Fiji, ImageJ 2.0.0-rc43/1.51s using colour deconvolution (1.7) for peroxidase staining, and then thresholded before the function “Analyse particles” was used to calculate the area fraction covered by P-gp.

### Data analysis

Data were analysed using MS Excel 2010 and SPSS v17. In addition to the filters built in the image analysis algorithm, filters were applied in data analysis software to eliminate situations with unacceptable image or staining quality. Those are described in S1 File Analysis of ARF data was performed with Prism 7 (GraphPad Software Inc.) using a nonparametric ANOVA Kruskal-Wallis test followed by an uncorrected Dunn’s test against the control. The test compared the individual sections to the control section of each animal.

## Supporting information

S1 Fig FUS treatment planning and brain sectioning. Illustration of FUS treatment planning and post-treatment brain tissue sectioning. a) Treatment plan for FUS exposure at 1.1 MHz, b) treatment plan for FUS exposure at 7.8 MHz, c) estimation of the ARF exposure area, d) brain sectioning schematics, coronal sections were used for ARF quantification in group 3 and horizontal sections were used for NP quantification in group 1, for histology in group 2 and P-gp evaluation in group 4.
(DOCX)

S2 Fig Size distribution of MBs. Size distribution of MBs used in our study.
(DOCX)

S1 Video Intracardiac injection of melanoma cells. Intracardiac injection of human melanoma cells in a NOD/SCID mouse, imaged by ultrasound.
(AVI)

S1 File Image analysis for determination of NP displacement from blood vessels. Image analysis of simulated images. Red–simulated NPs. Green–simulated blood vessels. Different scenarios (panel a-g), including NPs located inside blood vessels (panel c) and therefore excluded from displacement quantification. Quantification results are shown in Panel h.
Actual CLSM images are further exemplified and optimized in scenarios 1–8, also describing exclusion criterion.
(DOCX)

S2 File Quantitative data for individual mice. The quantitative data presented in Figs 4–6 are given in the excel file. Fig 4. MRI intensity and NP uptake and displacement data for individual mice. Fig 5. ARF induced displacement of NPs from blood vessels in individual mice and up to 10 frozen tissue sections. Fig 6. P-gp expression given as % area covered by P-gp staining in ROIs in individual mice and individual tissue sections.
(XLSX)
