# Supplementary material for: Ultrasound-mediated delivery and distribution of polymeric nanoparticles in the normal brain parenchyma of a metastatic brain tumour model
Source: PLoS One. 2018 Jan 16;13(1):e0191102. doi: 10.1371/journal.pone.0191102 (PMC5770053; doi:10.1371/journal.pone.0191102)
Supplement: S1 Fig — Illustration of FUS treatment planning and post-treatment brain tissue sectioning. a) Treatment plan for FUS exposure at 1.1 MHz, b) treatment plan for FUS exposure at 7.8 MHz, c) estimation of the ARF exposure area, d) brain sectioning schematics, coronal sections were used for ARF quantification in group 3 and horizontal sections were used for NP quantification in group 1, for histology in group 2 and P-gp evaluation in group 4. (DOCX) [file pone.0191102.s001.docx]

**Supplementary Fig 1** shows FUS treatment planning, both for FUS exposure at 1.1 MHz and for FUS exposure at 7.8 MHz, as well as a schematic of brain sectioning after brain removal and estimation of the ARF exposure area using an MR image. A grid of 6x2 circular regions each with a diameter of 1.6 mm were treated with FUS at 1.1 MHz **(panel a)** for BBB opening. An area of 2x2 regions each with a diameter of 0.5 mm was treated with FUS at 7.8 MHz for ARF **(panel b)**. The distance between this area and the top of the brain was measured in MR images as shown in **panel c**. Blue squares in panels **a-b** indicate the grid areas where FUS treatment started.


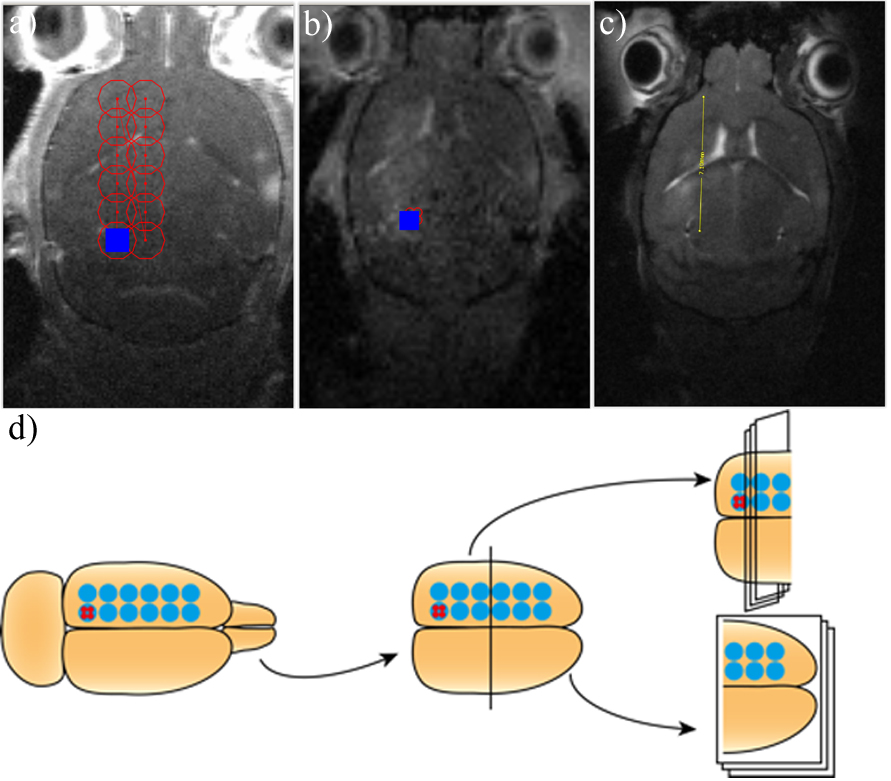
 **!**

**S1 Fig**  Illustration of FUS treatment planning and post-treatment brain sectioning. a) Treatment plan for FUS exposure at 1.1 MHz, b) treatment plan for FUS exposure at 7.8 MHz, c) estimation of the ARF exposure area, d) brain sectioning schematics, coronal sections were used for ARF quantification in group 3 and horizontal sections were used for NP quantification in group 1, for histology in group 2 and P-gp evaluation in group 4.
