# Supplementary material for: Ultrasound-mediated delivery and distribution of polymeric nanoparticles in the normal brain parenchyma of a metastatic brain tumour model
Source: PLoS One. 2018 Jan 16;13(1):e0191102. doi: 10.1371/journal.pone.0191102 (PMC5770053; doi:10.1371/journal.pone.0191102)
Supplement: S2 Fig — Size distribution of MBs used in our study. (DOCX) [file pone.0191102.s002.docx]

**Supplementary Fig2** shows a histogram with the size distribution of MBs used in this study.


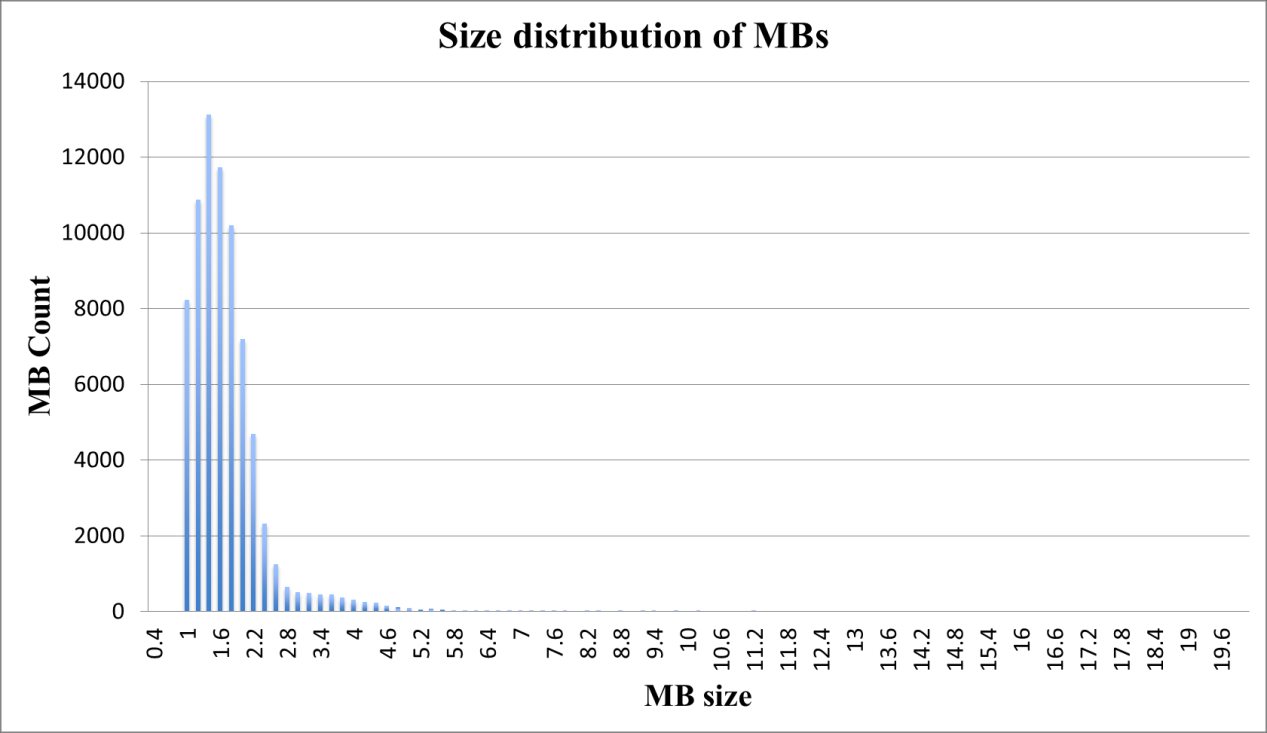


**S2 Fig.** Size distribution of MBs used in our study.
