# Supplementary material for: Ultrasound-mediated delivery and distribution of polymeric nanoparticles in the normal brain parenchyma of a metastatic brain tumour model
Source: PLoS One. 2018 Jan 16;13(1):e0191102. doi: 10.1371/journal.pone.0191102 (PMC5770053; doi:10.1371/journal.pone.0191102)
Supplement: S1 File — Image analysis of simulated images. Red–simulated NPs. Green–simulated blood vessels. Different scenarios (panel a-g), including NPs located inside blood vessels (panel c) and therefore excluded from displacement quantification. Quantification results are shown in Panel h. Actual CLSM images are further exemplified and optimized in scenarios 1–8, also describing exclusion criterion. (DOCX) [file pone.0191102.s004.docx]

Supplementary file 1

For NP quantification and assessment of their displacement away from blood vessels, images were thresholded and segmented using a three-level Otsu thresholding algorithm. Blood vessel segmentation uses a median filter and has built-in filters to remove obvious debris. NP segmentation was performed without any median filtering and relied on the quality of the raw images, although it also has built-in filters to remove artefacts (extremely large clumps). The reason is that additional filtering even with a small filtering radius could eliminate a significant number of NP. For determining NP displacement from blood vessels, NPs with a certain axis length, deemed to be inside blood vessels even where the corresponding blood vessel staining either failed or produced a signal that was too weak, were eliminated. Afterwards, NPs located outside of blood vessels were isolated by applying a sequence of image masks. Finally, the distance between NPs and the nearest blood vessels was found by object expansion until the neighboring pixels touched. Image analysis of simulated images and confocal image scenarios is shown below.

**Analysis of simulated images**


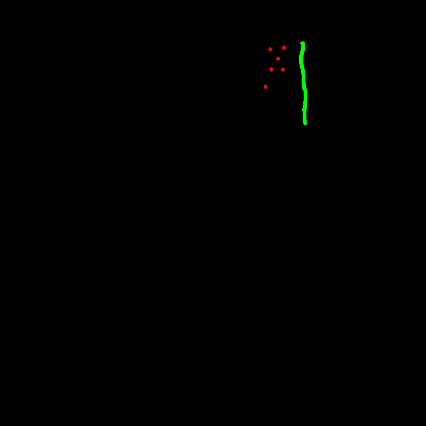

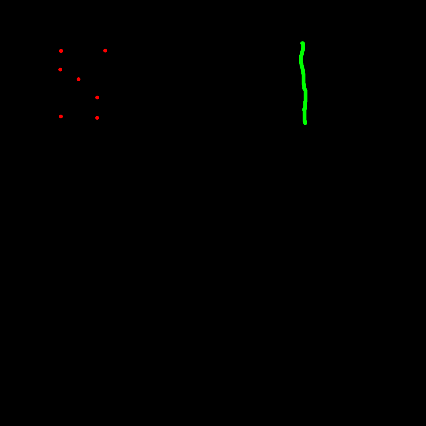

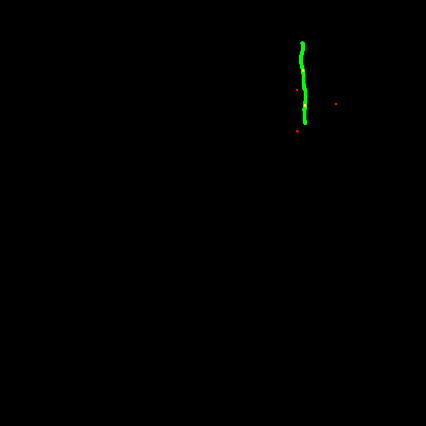


c)

b)

a)


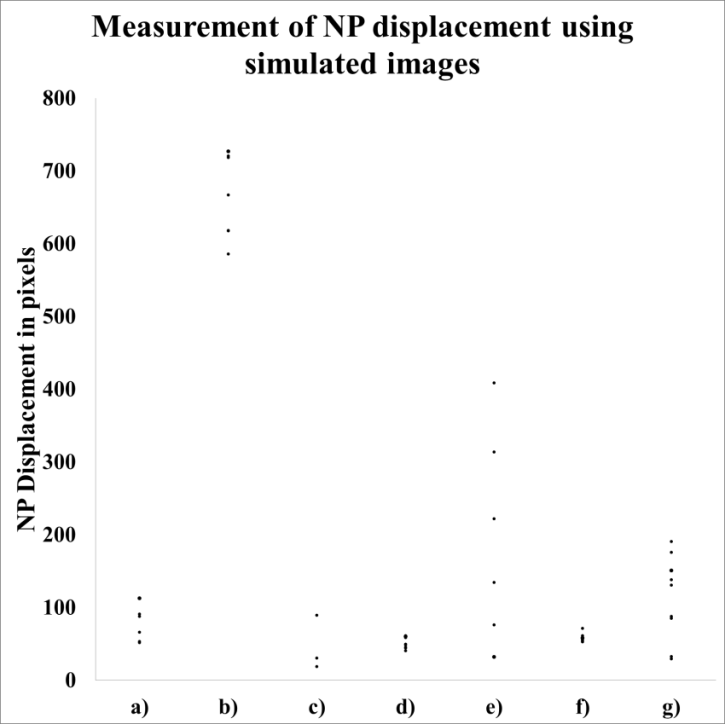

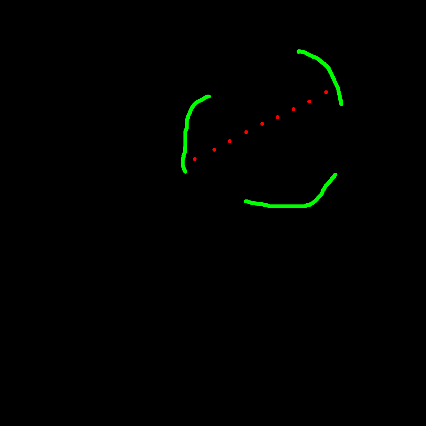

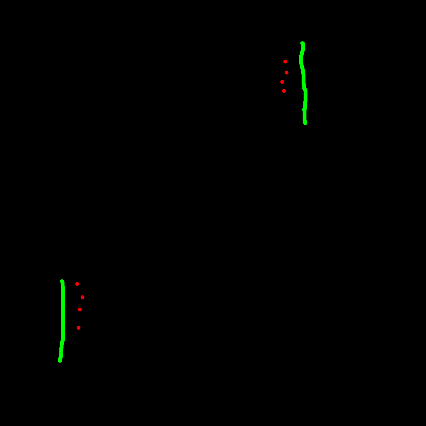

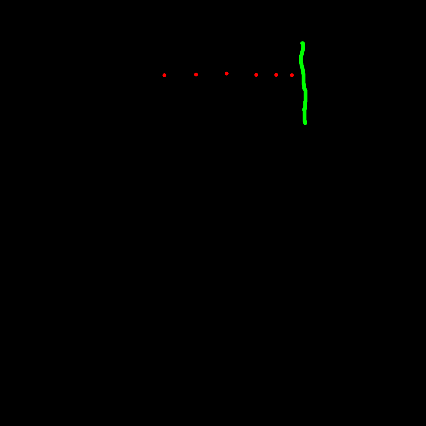

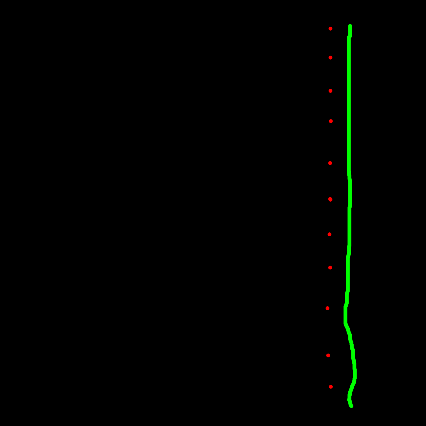


h))

d)

f)

e)

g)

Image analysis of simulated images.

Red – simulated NPs. Green –

Simulated blood vessels. Different

scenarios, including NPs

located inside blood vessels

(and, therefore, excluded

from displacement quantification – panel c) are shown in Panels a-g. Quantification results are shown in Panel h.

Shown below are several of the actual CLSM image scenarios used in algorithm training and optimization. For illustration purposes, scenarios are divided into four images. The first two images in a row are raw unprocessed images showing blood vessels and NPs, respectively. The 3^rd^ image shows segmented NPs overlaid on segmented blood vessels. In this image, NPs that are inside blood vessels are shown in blue (based the application of masks), and NPs outside of blood vessels are shown in red. No other filtering steps are applied in this image. The 4^th^ image is similar to the 3^rd^ except that NPs seemingly located outside of blood vessels have been additionally filtered to exclude those that are deemed to be inside blood vessels, even though staining of the corresponding blood vessel either failed or was too dim to be accurately segmented in a uniform segmentation applied to all images in the dataset. Additional filters applied on the image level are: **1)** blood vessel count over 50, **2)** blood vessel area between 2 and 12% of the image, **3)** number of NPs outside blood vessels over 3, and **4)** the ratio of the number of NPs located within 100 to 1000 pixels from the nearest blood vessel to the number of NPs located within 100 pixels from the nearest blood vessel less than 0.5 (this threshold substantially exceeds the average ratio of ≤0.1 and is set to adjust for all reasonable scenarios). These are referred to as **exclusion criteria** and were set on the basis of visual inspection of images in order to exclude images with poor staining or automatic segmentation quality.

**Scenario 1: Regular Scenario**


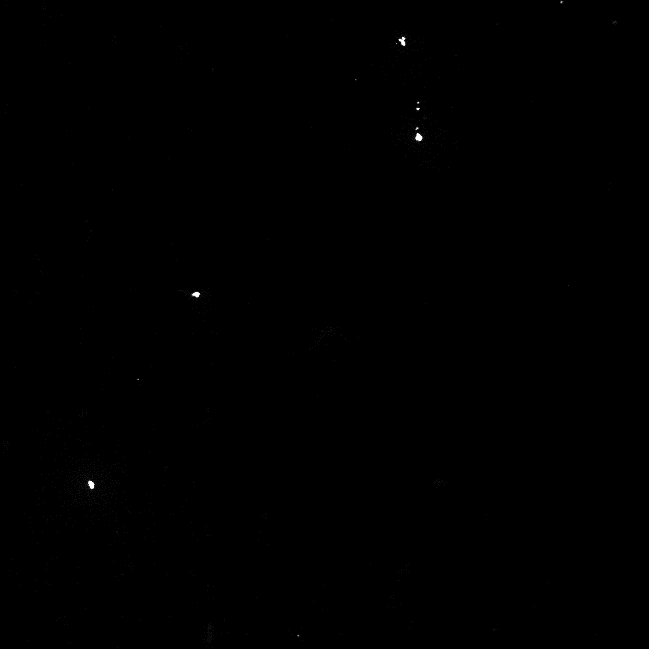

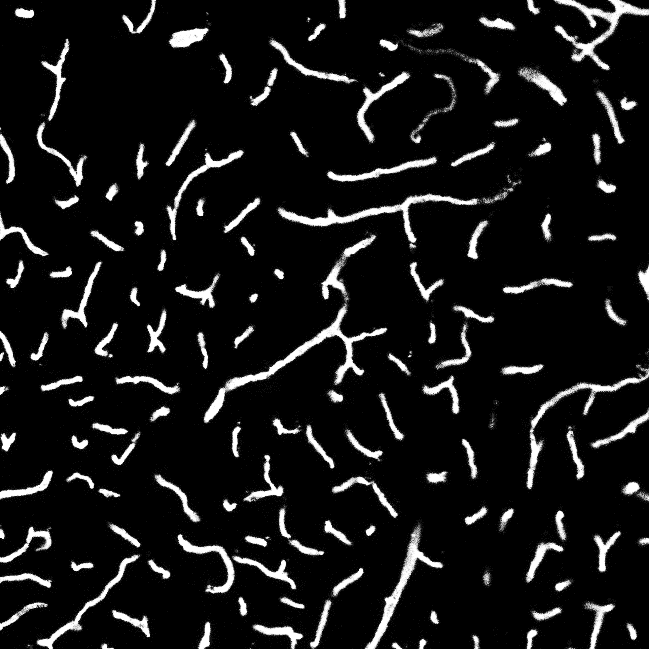

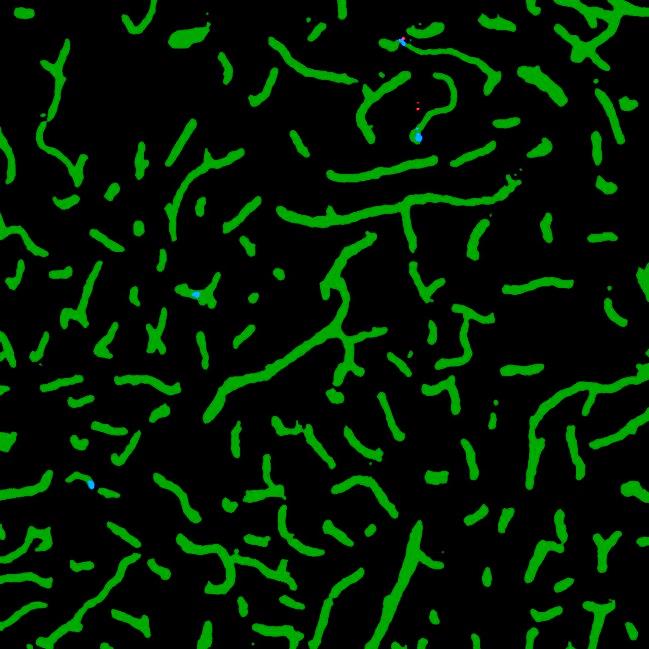

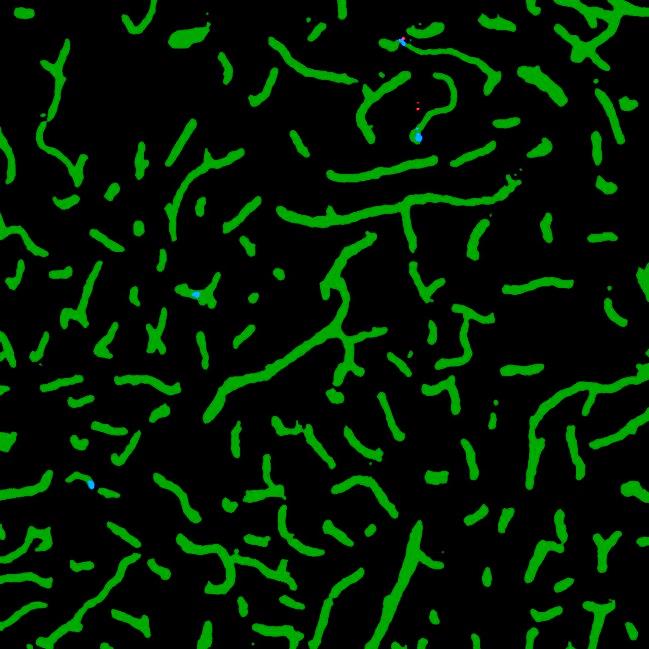


This is a good scenario showing NPs both inside and outside of blood vessels (indicated by red and blue arrows, respectively). No particular image analysis challenges are observed.

**Scenario 2: All NPs inside blood vessels**


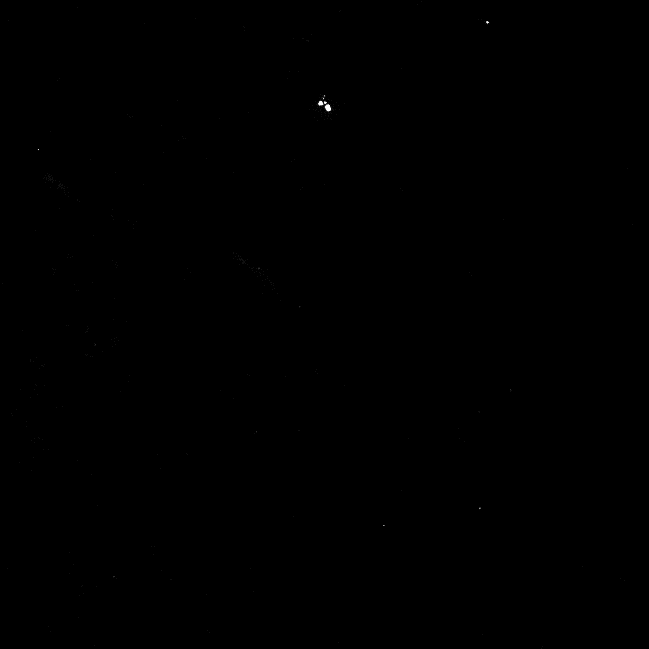

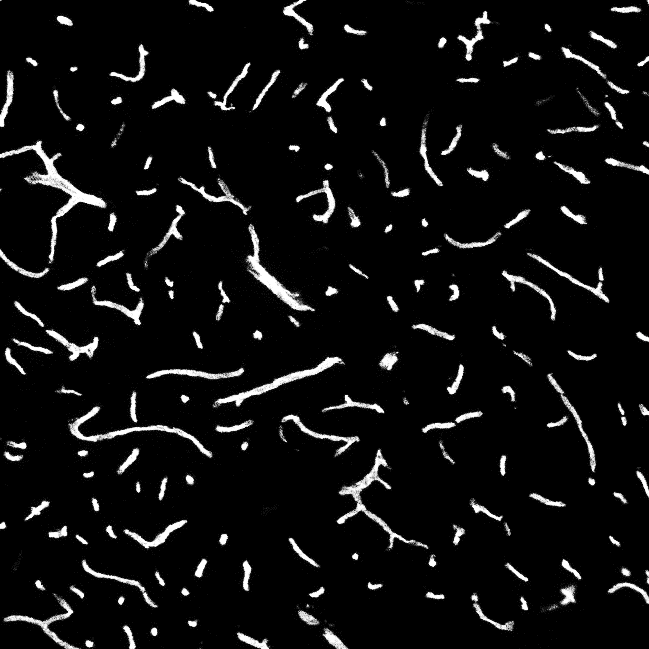


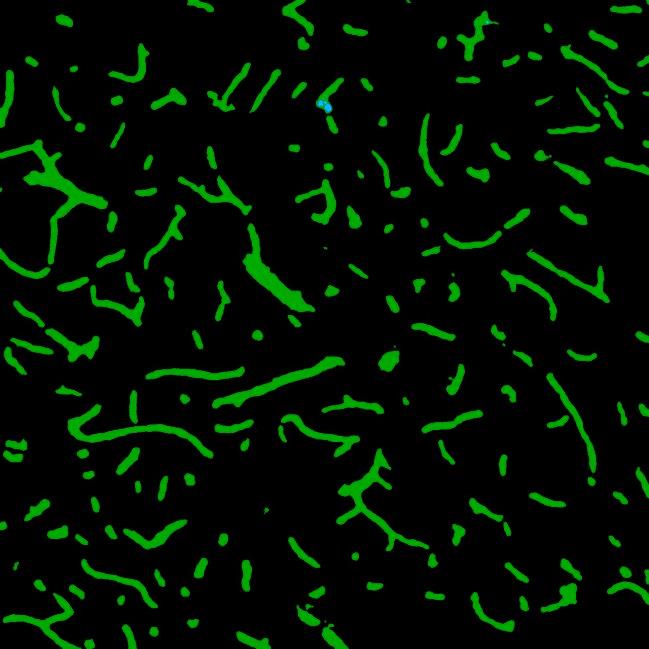

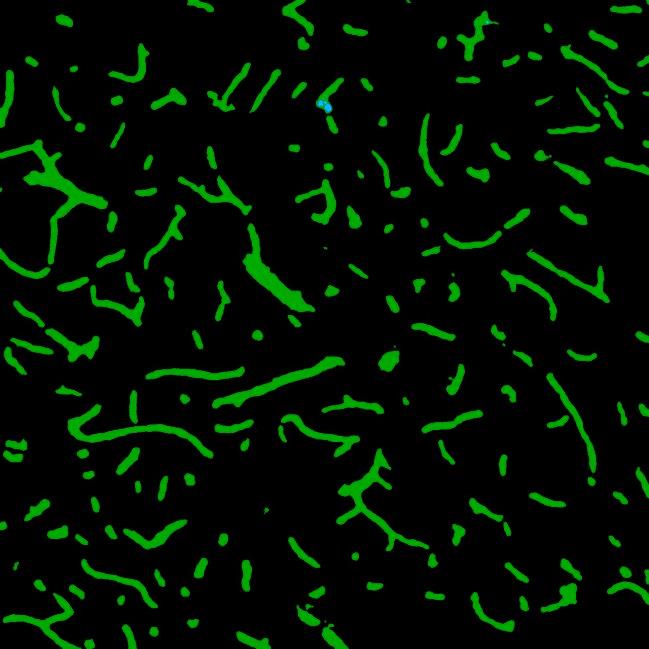


Again, a relatively easy scenario similar to Scenarios 1-2. Isolated spots are can still be detected inside of blood vessels (indicated by blue arrows), but the image fails the NP count exclusion criterion **(Exclusion Criterion 3).**

**Scenario 3: Unrealistic Blood Vessels**


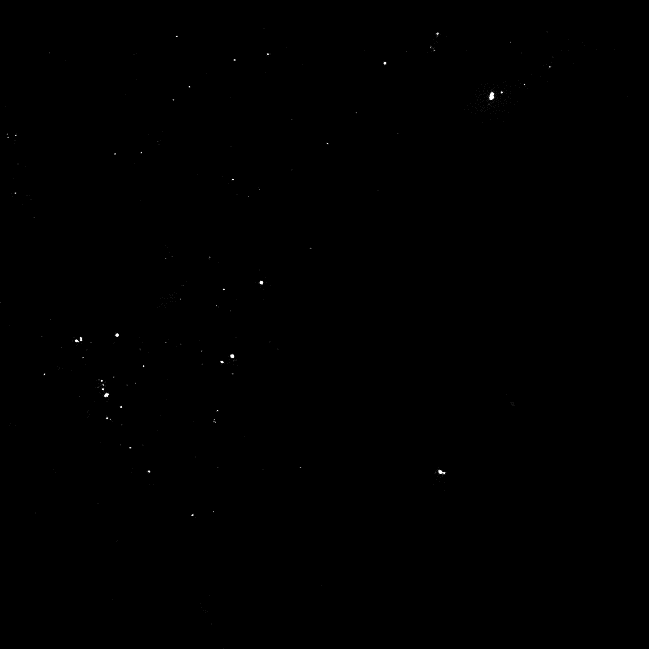

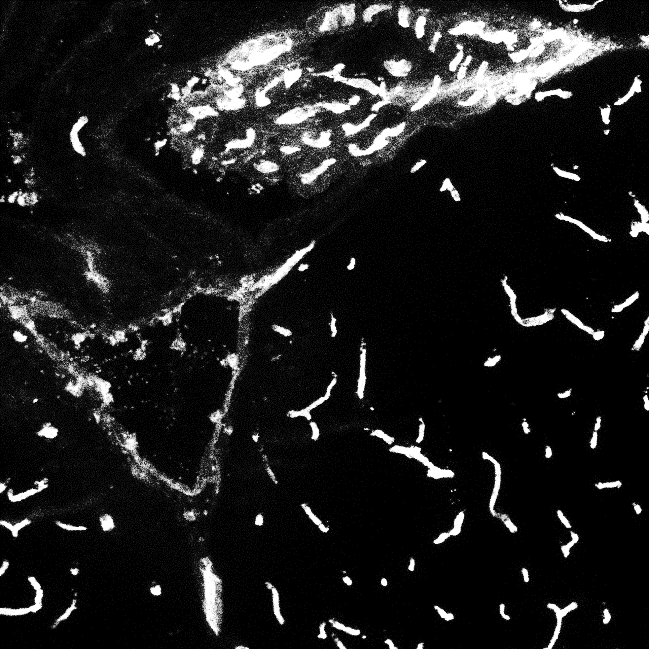


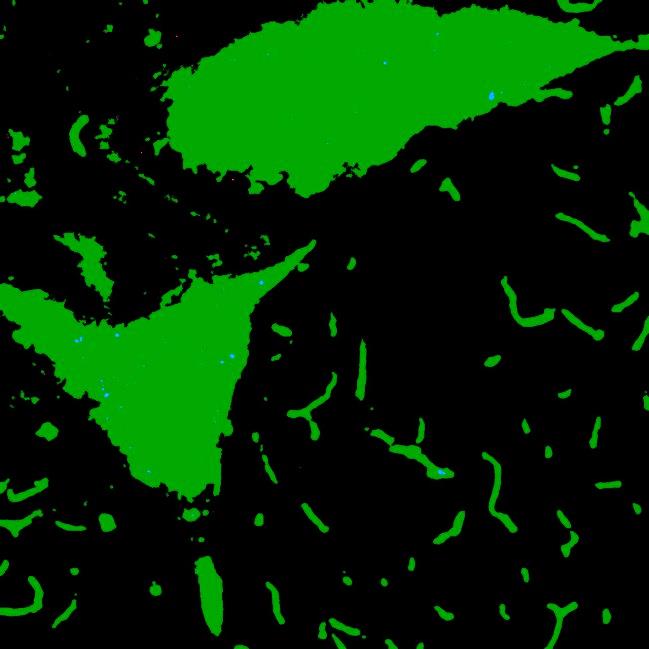

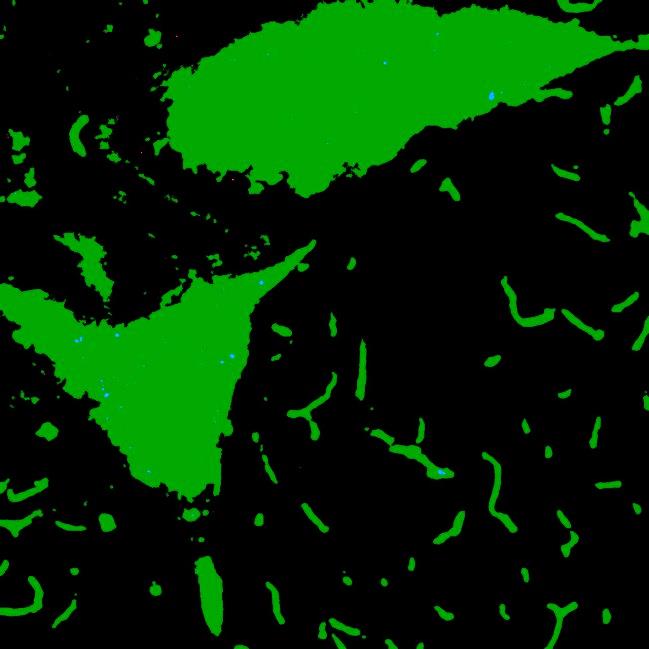


This scenario clearly shows an abnormal segmentation result. Filters could easily be applied to remove the two large ‘blood vessel’ clumps, but we preferred to filter out the entire image instead, based on the unreasonably large blood vessel area (25.5% of the image), i.e. this image failed **Exclusion Criterion 2**.

**Scenario 4: Dispersed NPs without blood vessels**


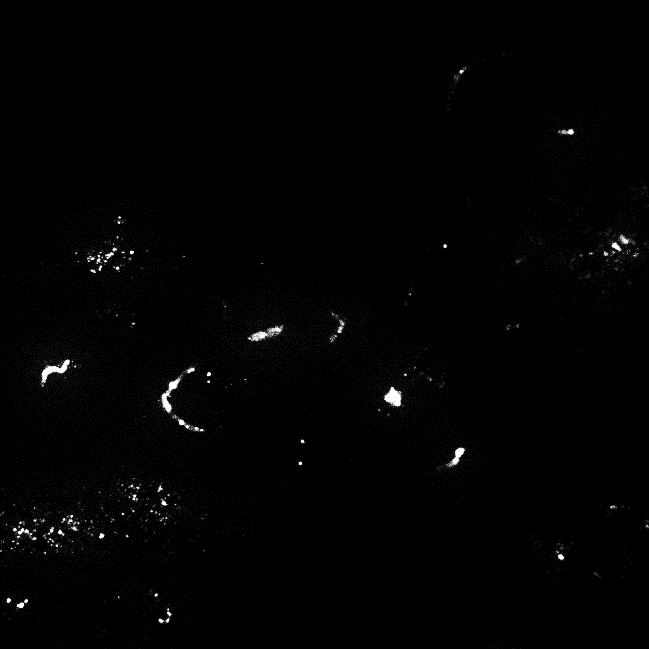

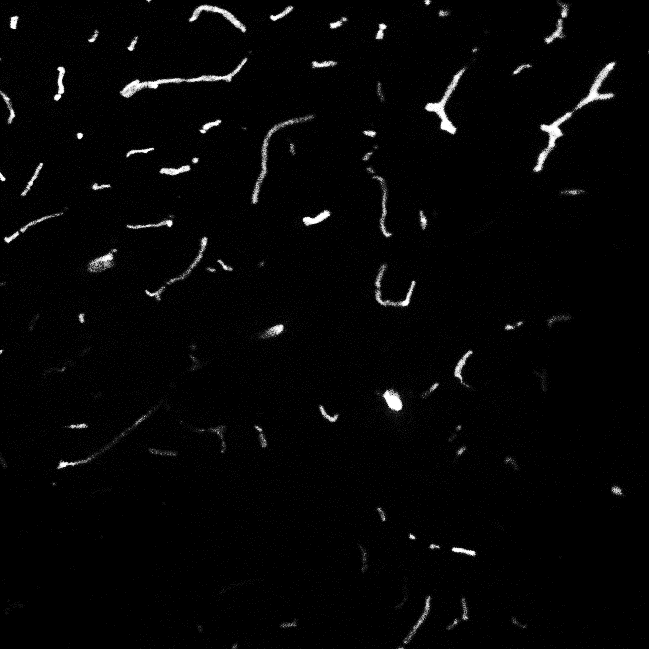


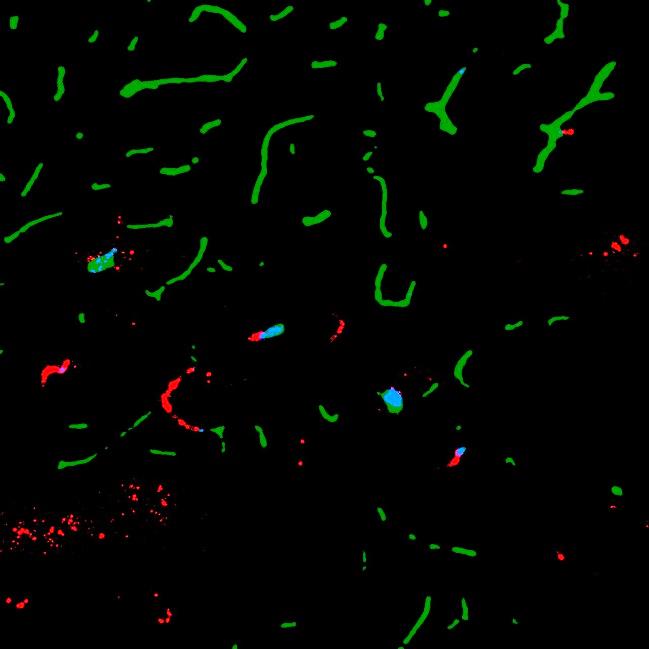

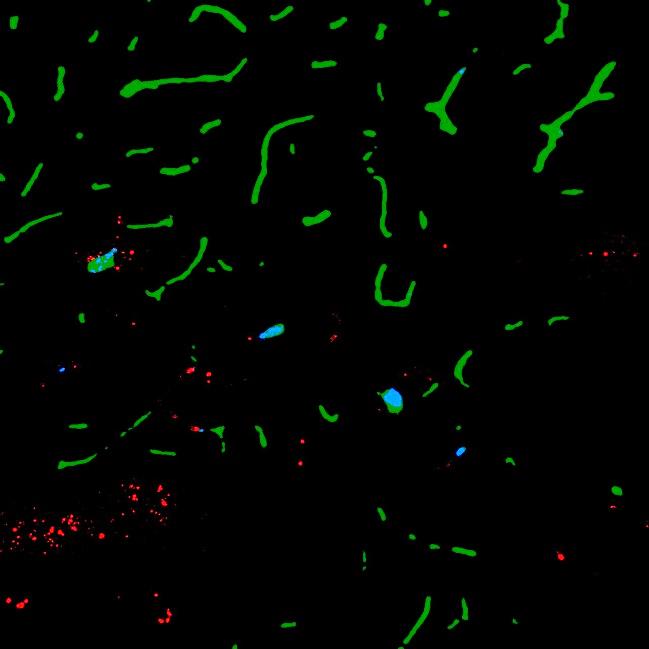


This is a relatively difficult scenario. Clumps of NPs that are clearly inside blood vessels, even though the corresponding blood vessel staining failed, can be filtered out easily (see below). However, we decided not to remove a cluster of NPs in the lower left corner even though they may or may not originate from a blood vessel that is not visible due to failed staining. This is both because of the lack of obvious exclusion criteria (no clear line connecting the NPs, unless one believes they have originated from a very thick blood vessel where the staining happens to have failed and the possibility that these NPs may have actually been displaced from the nearest visible blood vessel. In addition, the use of very sophisticated parameters (number of neighbors detected within a certain distance etc.) would considerably increase processing time and could affect segmentation in other images in a manner that can be difficult to predict, assuming uniform application of the algorithm throughout the entire dataset. However, this particular image is filtered out altogether, based on **Exclusion Criterion 4**. In this image, the ratio is 0.98, well above the average value. In addition, while this image does not fail **Exclusion Criterion 2**, it is very close to the criterion’s lower limit.

**Scenario 5: Clumped NPs**


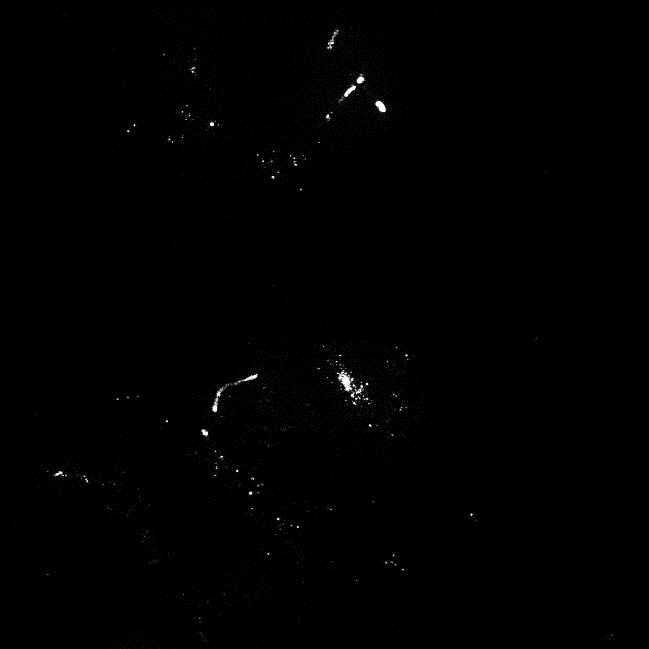

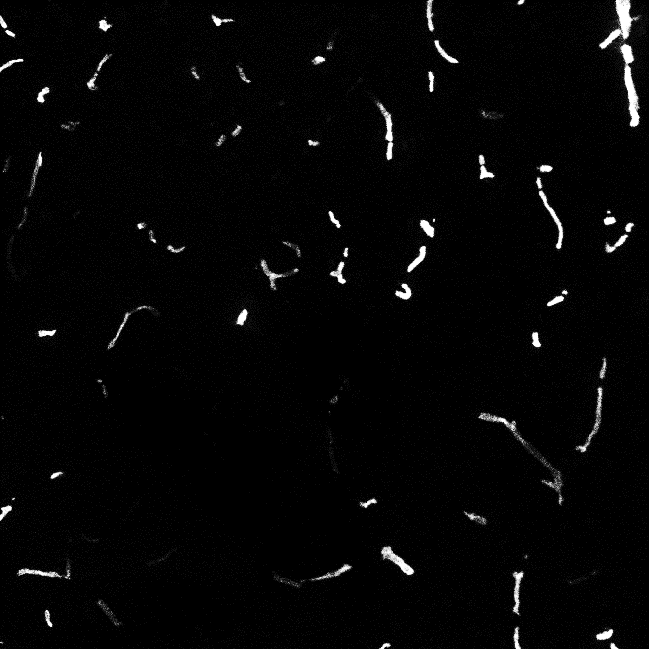


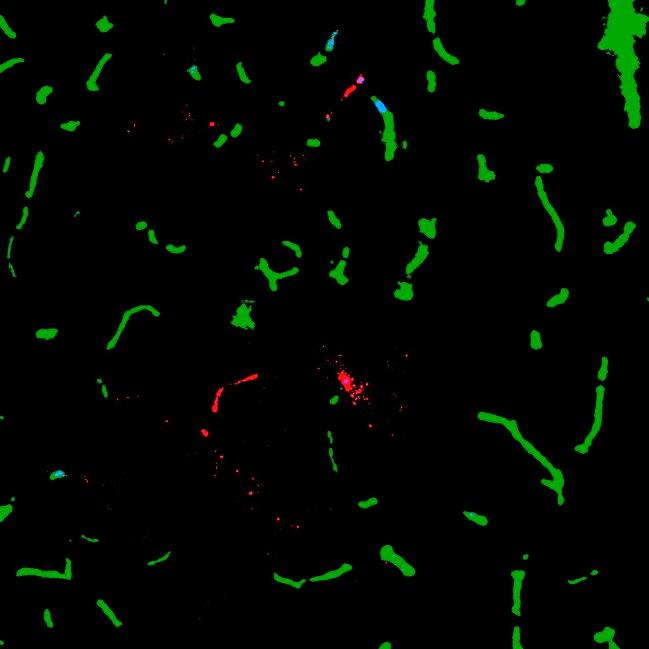

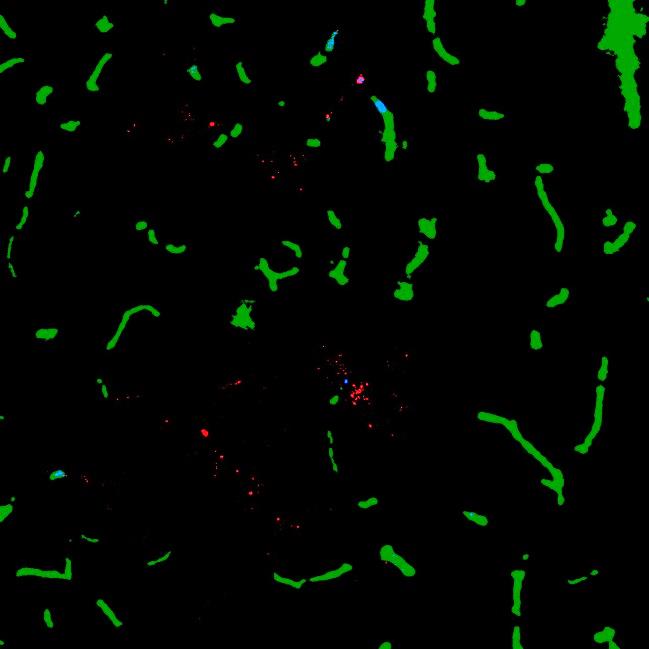


This is a fairly common case observed when there is a substantial number of NPs in an image. Some of the NPs in clumps are clearly in the blood vessels and are filtered by using axis length as a criterion. Setting the limit to exclude these and other similar clumps is a subjective call based on visual inspection of images, but the same criterion is applied to all images in the dataset. One can argue that the isolated group of the NPs located along a line (indicated by an arrow) should also be filtered. This is, however, not a trivial task to be performed consistently in automated image analysis.

**Scenario 6: Few NPs, mostly inside blood vessels**


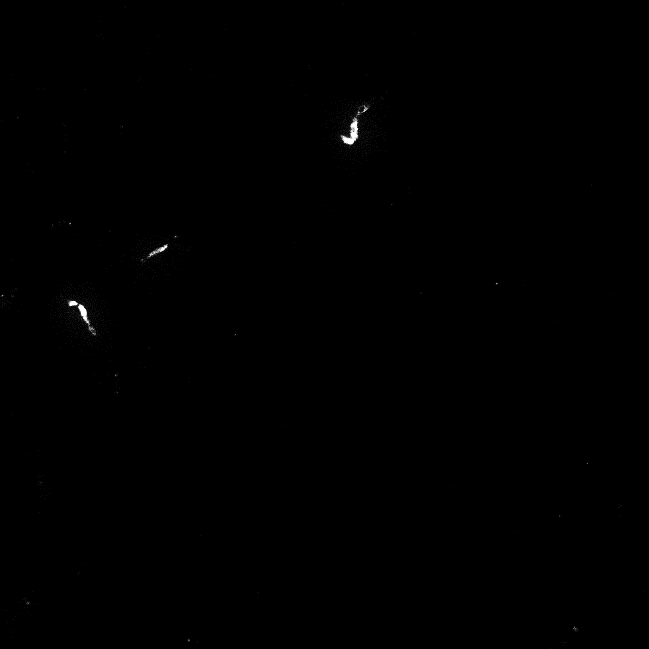

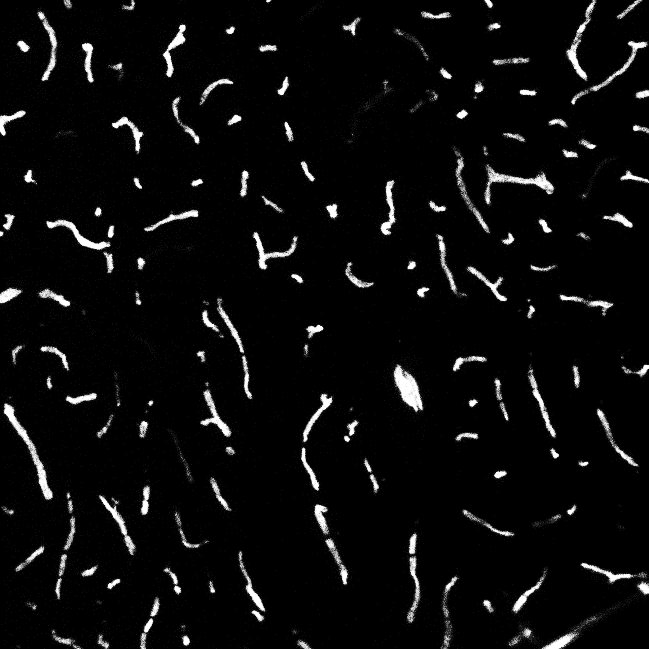


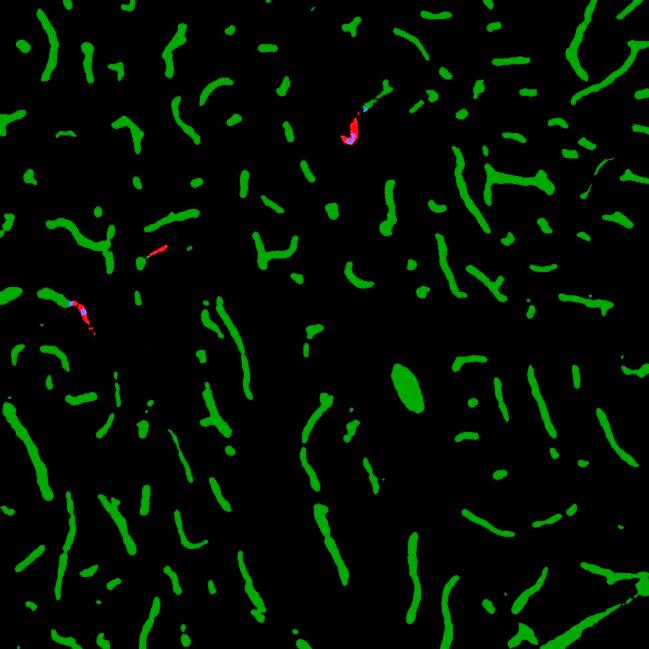

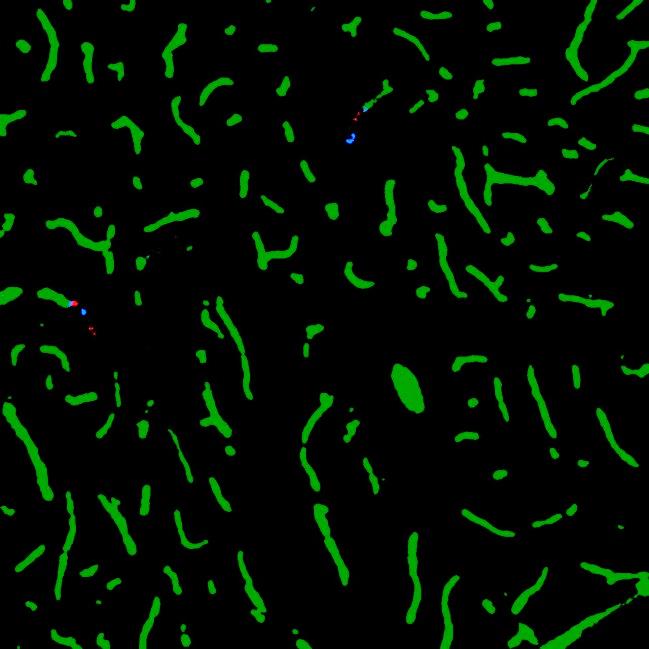


A relatively easy scenario, mostly shown to illustrate filtering or NPs that are deemed to be inside their adjacent blood vessels (indicated by an arrow).

**Scenario 7: Clustered and elongated NPs**


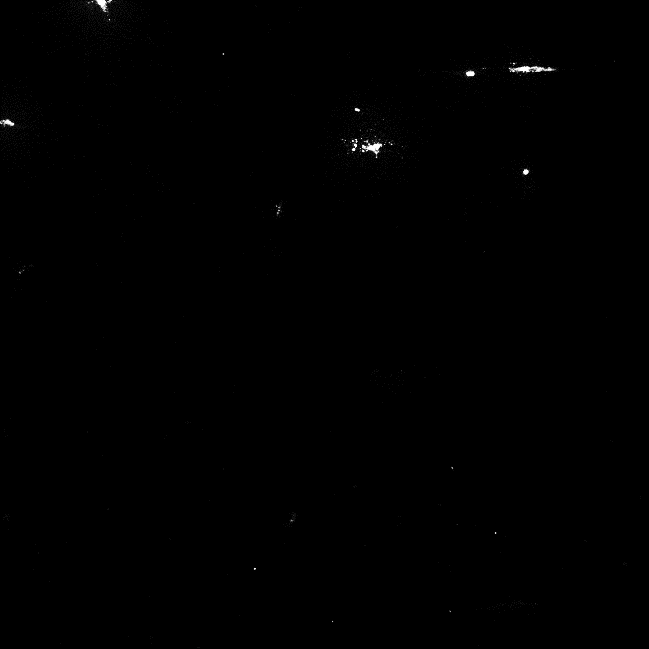

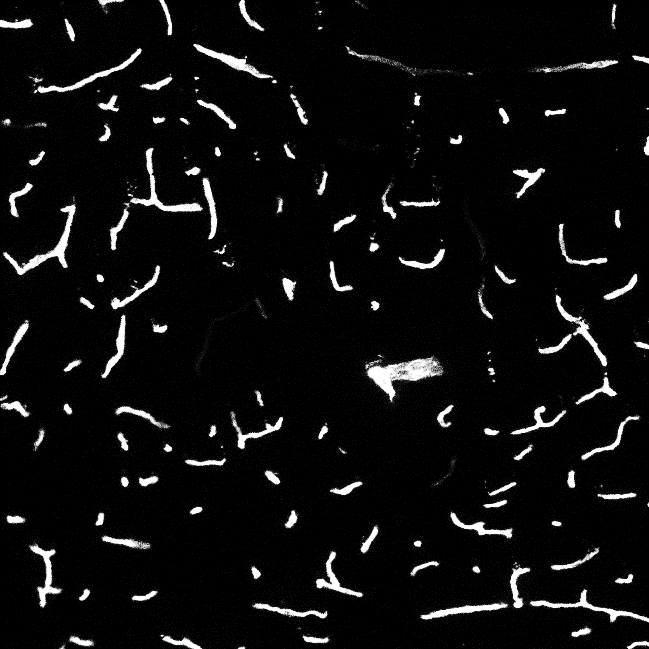


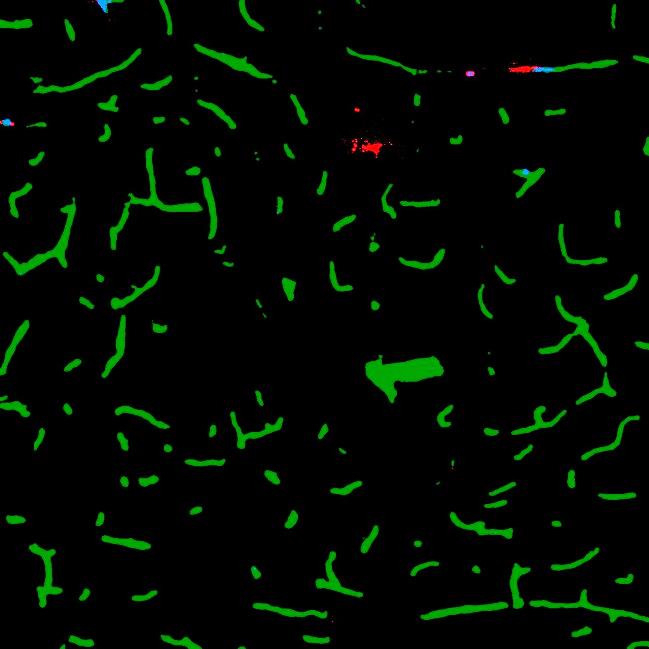

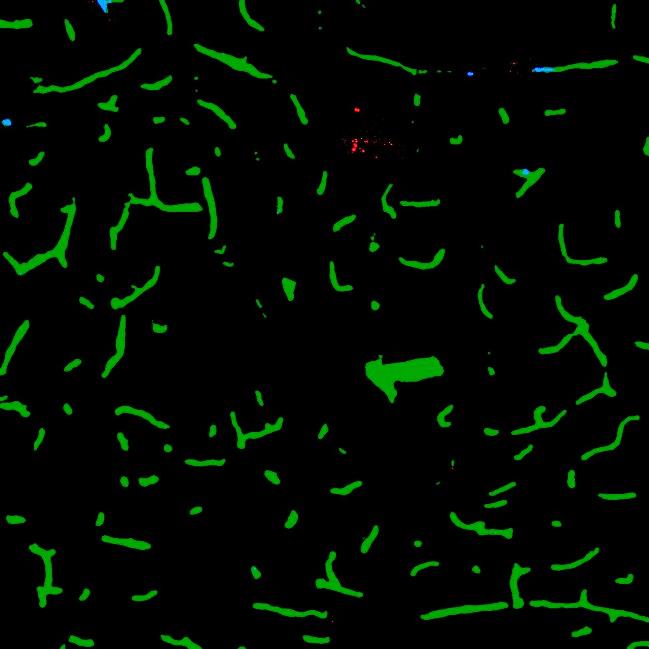


This scenario shows both clustered and elongated NPs. The group of NPs indicated by an arrow most likely originates from a blood vessel, and the central part is filtered out, although the dispersed cloud is retained. This is similar to Scenario 5.

**Scenario 8: Very few blood vessels with poor staining, large spread of NPs**


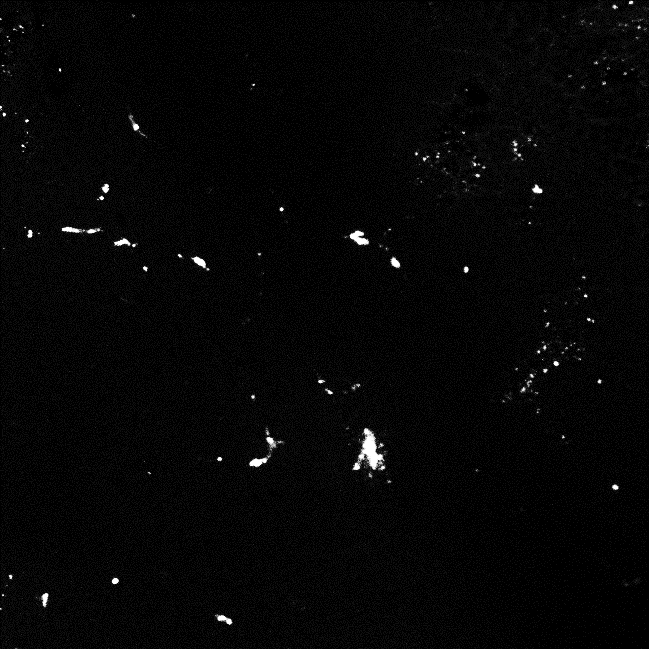

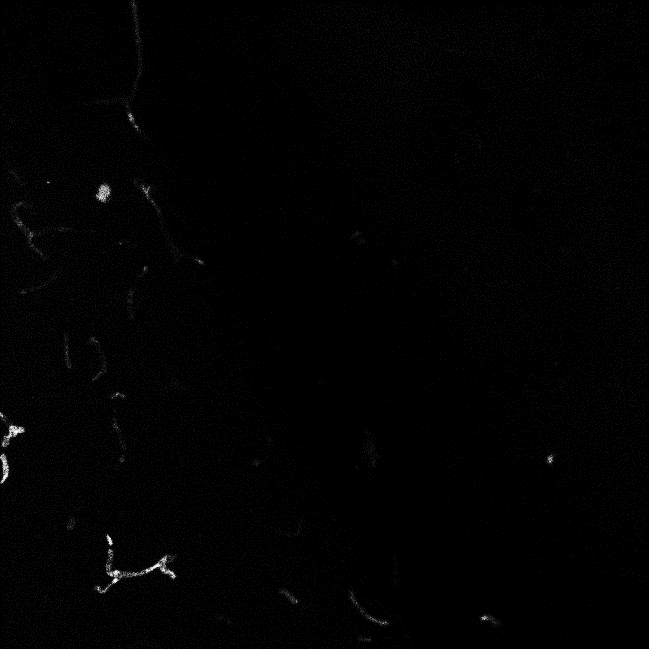


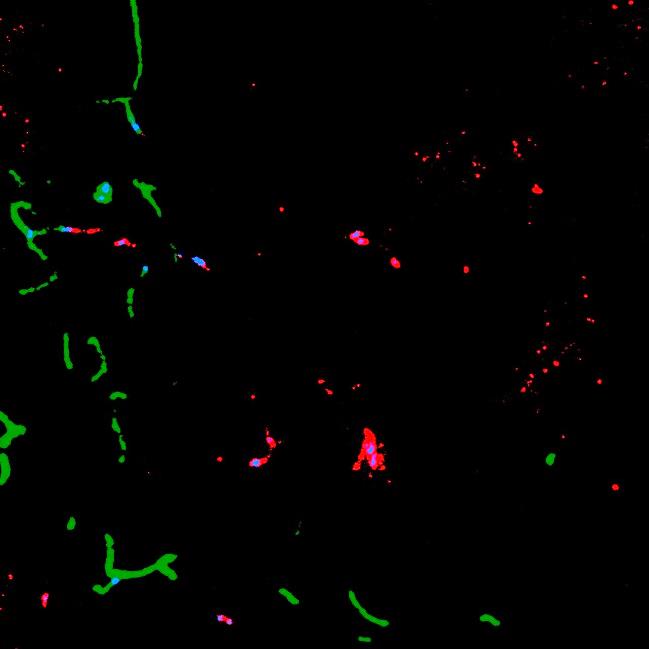

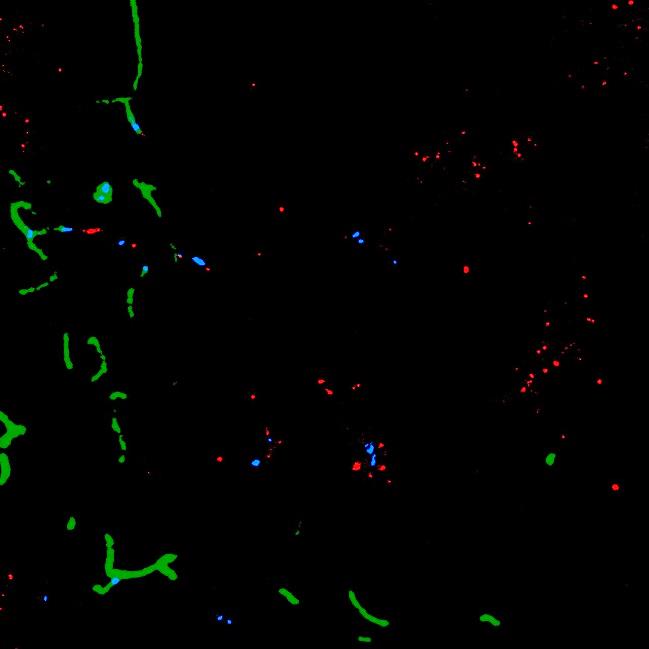


This is a relatively easy scenario. While the algorithm segments blood vessels rather well, given poor staining quality, this image fails several exclusion criteria at once (total area of blood vessels in the image being too low, number of segmented blood vessels being too low, and the ratio of NPs located within 100 to 1000 pixels from the nearest blood vessel to NPs located within 100 pixels from the nearest blood vessel being too high.
